# Yougui Pills Alleviate Osteoporosis by Inhibiting Mesenchymal Stem Cell ROS Accumulation via the Nrf2/HO‐1 Pathway

**DOI:** 10.1111/jcmm.71295

**Published:** 2026-07-22

**Authors:** Jiangyuan Liu, Kairui Chen, Jingyuan Wen, Qinghe Zeng, Liangyan Cheng, Qinwen Ge, Jiali Chen, Wenhua Yuan, Pinger Wang, Luwei Xiao, Hongting Jin, Yungang Wu

**Affiliations:** ^1^ Institute of Orthopaedics and Traumatology of Zhejiang Province Hangzhou Zhejiang China; ^2^ The First College of Clinical Medicine Zhejiang Chinese Medical University Hangzhou Zhejiang China; ^3^ The First Affiliated Hospital of Zhejiang Chinese Medical University (Zhejiang Provincial Hospital of Chinese Medicine) Hangzhou Zhejiang China; ^4^ College of Pharmaceutical Sciences Zhejiang Chinese Medical University Hangzhou China; ^5^ Department of the Orthopedics of TCM First Affiliated Hospital of Wenzhou Medical University Wenzhou Zhejiang Province China

**Keywords:** mesenchymal stem cells, Nrf2/HO‐1 pathway, osteoporosis, oxidative stress, traditional chinese medicine, yougui pills

## Abstract

Osteoporosis (OP) is a prevalent skeletal disorder characterized by progressive bone mass loss and deteriorated microarchitecture, in which oxidative stress‐induced mesenchymal stem cell (MSC) dysfunction serves as a core pathogenic mechanism. Yougui Pills (YGPs), a classical traditional Chinese medicine formula, are widely applied in clinical OP management, yet the cellular and molecular mechanisms underlying their anti‐osteoporotic effects remain incompletely defined. This study aimed to elucidate the bone‐protective role of YGPs in OP and explore the underlying mechanism, focusing on oxidative stress modulation in MSCs and the Nrf2/HO‐1 signalling pathway. The bioactive components of YGPs and YGP‐containing serum were characterized via UPLC. In vivo, an ovariectomy (OVX) mouse model was established to evaluate YGPs' effects on bone mass, trabecular microstructure and osteogenesis using micro‐CT, histological and immunohistochemical assays; public GEO datasets were re‐analysed to profile transcriptomic alterations and oxidative stress signatures in OP‐derived MSCs. In vitro, H_2_O_2_ was used to induce ROS accumulation and oxidative injury in MSCs, with assessments of cell proliferation, apoptosis, ROS levels, migration and osteogenic differentiation. Network pharmacology and siRNA‐mediated gene silencing were conducted for target prediction and mechanistic validation. In vivo results showed that YGP treatment significantly ameliorated OVX‐induced osteopenia, increased bone mineral density, improved trabecular microstructure, and upregulated osteogenic markers (ALP, OCN, Runx2, COL1A1). Transcriptomic re‐analysis revealed upregulated oxidative stress markers in OP MSCs, consistent with In vitro findings that YGPs attenuated H_2_O_2_‐triggered ROS overproduction, suppressed apoptosis and ectopic lipid deposition, and enhanced MSC proliferation, migration and osteogenic differentiation. Mechanistically, YGPs activated the Nrf2/HO‐1 signalling axis, while Nrf2 knockdown abrogated YGPs' cytoprotective and pro‐osteogenic effects. In conclusion, YGPs mitigate oxidative stress‐induced MSC dysfunction and promote osteogenesis via the Nrf2/HO‐1 pathway, supporting YGPs as a promising therapeutic candidate for OP.

**Abbreviations**
ABH/OG: Alcian Blue Haematoxylin/Orange G
ALP: Alkaline Phosphatase
ARS: Alizarin Red S
BMD: bone mineral density
BV/TV: bone volume fraction
GAPDH: glyceraldehyde‐3‐phosphate dehydrogenase
GEO: Gene Expression Omnibus
HE: Haematoxylin and Eosin
HO‐1: heme oxygenase‐1
IF: Immunofluorescence
IHC: Immunohistochemistry
MSCs: mesenchymal stem cells
NAC: N‐acetylcysteine
NOX2: NADPH oxidase 2
Nrf2: nuclear factor erythroid 2‐related factor 2
OCN: Osteocalcin
OP: osteoporosis
Osterix: Sp7 transcription factor 7
OVX: ovariectomized
ROS: reactive oxygen species
Runx2: runt related transcription factor 2
Tb. N: trabecular number
Tb. Sp: trabecular separation
Tb. Th: trabecular thickness
TCM: Traditional Chinese medicine
UPLC: Ultra‐Performance Liquid Chromatography
YGPs: Yougui pills
β‐actin: actin, beta

## 1 Introduction

Osteoporosis (OP), a widespread metabolic bone disorder affecting millions globally, manifests through progressive bone mass reduction and microarchitectural deterioration [1]. OP affects > 200 million people globally, with prevalence rising steeply with age; estimates reach 21.2% in women and 6.3% in men worldwide and up to 49% and 23% in China, respectively [2, 3]. Hip and vertebral fractures drive pain, disability, and excess mortality, imposing major socioeconomic costs [4]. Pathophysiologically, OP reflects an imbalance between bone resorption and formation [5]. Although antiresorptives (e.g., bisphosphonates, denosumab) reduce fracture risk, they face adherence and safety limitations and may oversuppress remodelling, delaying improvements in bone quality [6, 7, 8]. These constraints highlight the need for mechanism‐based strategies that not only inhibit resorption but also rebuild bone and microarchitecture, including osteoanabolic approaches.

Mesenchymal stem cells (MSCs) are nutritionally regulated key players in skeletal homeostasis, serving as the progenitor pool for osteoblast lineage cells and coordinating bone remodelling via nutrition‐modulated osteo‐immune crosstalk and paracrine signalling [9, 10]. In OP, aging and oestrogen deficiency disrupt the marrow's nutritional microenvironment, driving chronic low‐grade inflammation and redox imbalance—both of which are closely linked to impaired nutritional metabolism—ultimately leading to excessive accumulation of reactive oxygen species (ROS) [11, 12]. Elevated ROS disrupts nutrient‐dependent mitochondrial bioenergetics, induces DNA damage, activates stress kinases, and suppresses nutrition‐sensitive pro‐osteogenic pathways (e.g., Wnt/β‐catenin). This further skews MSCs lineage commitment toward adipogenesis at the expense of osteogenesis—an imbalance exacerbated by suboptimal nutrient supply—thereby increasing marrow adiposity and reducing osteoblastogenesis [13, 14, 15]. Concomitantly, ROS impairs MSCs proliferation, migration, and differentiation—key processes supported by adequate nutritional cues—collectively accelerating trabecular deterioration and bone loss [16]. Thus, strategies that limit ROS accumulation, restore nutrient‐dependent mitochondrial quality control, and re‐establish MSCs osteogenic competence represent rational, nutrition‐aligned, mechanism‐based interventions for OP prevention and management.

Traditional Chinese Medicine (TCM) provides a rich reservoir for anti‐osteoporotic discovery, with several botanicals reported to modulate MSCs function and osteogenesis (e.g., *Epimedii Folium* via Wnt/β‐catenin; *Davallia trichomanoides Blume* influencing oestrogen levels and trabecular density) [17, 18, 19]. Osteoporosis belongs to the categories of “bone atrophy” or “bone impediment” in TCM, with its core pathogenesis being deficiency of Kidney essence and insufficiency of Kidney‐Yang due to aging, leading to insufficient marrow production and bone malnutrition. According to the TCM theory that “the Kidney governs bones and produces marrow”, the Kidney stores essence, which generates marrow, and marrow nourishes bones. The growth, development, and health of bones depend on the sufficiency of Kidney essence. Yougui Pills (YGPs), a classical formula first documented in *Jingyue Quanshu* (Ming Dynasty) by Zhang Jingyue, is a representative prescription for “tonifying Kidney‐Yang” in TCM. Its formulation follows the TCM principle that “those who are good at tonifying Yang must seek Yang within Yin, so that Yang receives Yin assistance and generates infinitely”. The monarch herbs (Fu zi, Rou gui, and Lu jiao jiao) warm and strengthen the primordial Yang and replenish essence and marrow. The minister herbs (Shu di huang, Shan yao, Shan zhu yu, and Gou qi zi) nourish Yin and tonify the kidney, liver, and spleen, embodying the essence of “seeking Yang within Yin”. The assistant herbs (Tu si zi, Du zhong, and Dang gui) tonify the liver and kidney, strengthen the waist and knees, and nourish and harmonize blood. Together, they exert the effects of warming and tonifying Kidney‐Yang and replenishing essence and marrow [20, 21]. Despite growing clinical experience and preclinical evidence supporting bone health, definitive mechanisms underlying YGPs' regulation of MSCs osteogenesis and oxidative stress remain insufficiently defined. Establishing rigorous chemistry–biology connections and validating these target pathways are therefore critical to integrating YGPs into contemporary, mechanism‐based intervention strategies for bone health.

Here, we hypothesized that YGPs alleviate OP by reducing ROS accumulation and restoring MSCs' osteogenic competence. To verify this hypothesis, we integrated In vivo animal studies and In vitro H_2_O_2_‐induced oxidative stress assays, along with network pharmacology and siRNA validation, to identify redox response targets and elucidate the bone‐protective mechanism of YGPs.

## 2 Material and Methods

### 2.1 Drugs

Yougui Pills are composed of ten traditional Chinese medicines (Table 1) and was purchased from Beijing Tongrentang Pharmaceutical Co. Ltd. (Batch number: Z11021040). Fosamax was purchased from Savio Industrial S.r.L (Italy). The antioxidant agent N‐acetylcysteine (NAC, Catalogue: S1623) was obtained from Selleck Chemicals (Shanghai, China).

**TABLE 1:** The compositions of Yougui pills.

Chinese name	Latin name	Family	Parts used	Weight (g)
Shu di huang	Rehmanniae radix praeparata	*Rehmannia glutinosa* (Gaertn.) DC.	Root	240
Shan yao	Dioscoreae rhizoma	*Dioscorea oppositifolia* L.	Rootstock	120
Shan zhu yu	Corni fructus	*Cornus officinalis* Siebold & Zucc.	Fruit	90
Gou qi zi	Lycii fructus	*Lycium barbarum* L.	Fruit	120
Lu jiao jiao	Cervi cornus colla	*Cervus elaphus* Linnaeus	Horn	120
Tu si zi	Cuscutae semen	*Cuscuta chinensis* Lam.	Fruit	120
Du zhong	Eucommiae cortex	*Eucommia ulmoides* Oliv.	Bark	120
Dang gui	Angelicae sinensis radix	*Angelica sinensis* (Oliv.) Diels	Root	90
Rou gui	Cinnamomi cortex	*Cinnamomum cassia* (L.) J.Presl	Bark	60
Fu zi	Aconiti lateralis radix preparata	*Aconitum carmichaelii* Debeaux	Root	60

The plant names have been checked with http://www.worldfloraonline.org.

### 2.2 Analysis of the Bioactive Components of YGPs

The YGPs and YGPs‐containing drug serum were analysed by ultra‐performance liquid chromatography coupled with quadrupole time‐of‐flight mass spectrometry (UPLC‐Q‐TOF/MS). The experimental procedures and parameters of ultra‐performance liquid chromatography (UPLC) were referred to our previous study [22]. The components separated by UPLC should be detected by tandem quadrupole time‐of‐flight mass spectrometry. Subsequently, the data were analysed using UNIFI V1.8 software.

### 2.3 Animals

Thirty 9‐week‐old female C57BL/6J mice (average weight 20 ± 2 g) were used in this investigation. Following institutional guidelines, all rodents underwent a 7‐day acclimation period with ad libitum access to food and water in SPF‐grade housing conditions featuring controlled 12‐h photoperiod cycles. All animal experiments in this study were conducted following National Institutes of Health (NIH) standards for animal experimentation and approved by the Experimental Animal Ethics Committee of the First Affiliated Hospital of Wenzhou Medical University (WYYY‐AEC‐YS‐2023‐0085). The number of the quality certificate of the laboratory animals is SCXK (Hu) 2023–0009.

### 2.4 Model Establishment and Treatment

The animals were randomly allocated into five experimental cohorts: sham‐operated controls (Sham), ovariectomy‐induced osteoporotic model (OVX), low‐dose YGPs group (YGPs‐Low, 1.75 g/kg), high‐dose YGPs group (YGPs‐High, 3.5 g/kg), and Fosamax group (Fosamax, 10.51 mg/kg). The YGPs dosage for mice was derived from the clinically effective human dose and adjusted for the surface area differences between humans and mice. Mice were anaesthetised via i.p. Injection of Telazol at 50 mg/kg. Following established protocols, bilateral ovary removal was performed on 10‐week‐old subjects (excluding sham controls) to create the OP model [23]. All groups received their respective drugs orally for 8 weeks, except the Fosamax group, which was treated weekly. The remaining groups were treated daily. Upon completion of the treatment protocol, euthanasia was administered using carbon dioxide.

### 2.5 Micro‐CT Assessment

The mice femur samples were fixed in 4% polyformaldehyde for 48 h, following which they were subjected to Micro‐CT (Skyscan1176, Bruker, Belgium) scanning. The scanning parameters are as follows: Source Voltage (kV) = 45, Source Current (uA) = 500, Filter = Al 0.2 mm, Rotation Step (deg) = 0.300. Image reconstruction software (NRecon) was used to reconstruct the images obtained from the scans, and the reconstructed data was then imported into the data viewer software to select the sites for analysis. The CTAn software was used to analyse various parameters of the femur and the number of slices selected was 100. Parameters analysed included: trabecular bone mineral density (Tra. BMD), trabecular number (Tb. N), bone volume fraction (BV/TV), trabecular thickness (Tb. Th) and trabecular separation (Tb. Sp). CTvox software was used to construct the 3D display images.

### 2.6 Tissue Staining, Immunohistochemistry and Immunofluorescence Staining

All samples were decalcified by soaking in 14% ethylenediaminetetraacetic acid solution for 2 weeks. The decalcified samples were rinsed with pure water and then placed in a dehydrator for programmed dehydration and paraffin embedding. Four‐micrometre‐thick coronal sections were cut from the distal end of the femur. These tissue slices initially underwent sequential staining with haematoxylin–eosin (HE) and Masson's trichrome techniques before being cover slipped for microscopic examination. According to the experimental protocol, immunohistochemistry (IHC) and immunofluorescence (IF) analyses were performed. During the pre‐staining preparation, histological sections were exposed to 0.3% Triton X‐100 solution for membrane permeabilization (10 min at ambient temperature). Subsequently, they were treated with an endogenous peroxidase inhibitor (ZSGB‐BIO, PV‐6001) for 20 min under standard laboratory conditions and then blocked with goat‐derived serum. Tissue sections received overnight incubation (12–16 h at 4°C) with the specified primary antibodies. The experimental analysis focused on biomarkers including ALP, OCN, Runx2, CD90, COL1A1, NOX2, Nrf2, and HO‐1 (Table 2). In the immunohistochemistry protocol, the appropriate secondary antibody was incubated for 20 min the next day. The antigen–antibody complexes were visualized with diaminobenzidine (DAB; ZSGB‐BIO, ZLI‐9018), and the nuclei were counterstained with haematoxylin. For fluorescence‐based detection, tissue sections underwent overnight primary antibody incubation followed by 30‐min exposure to fluorophore‐conjugated secondary antibodies the subsequent day. The nuclei were counterstained with DAPI for 10 min. The processed specimens were then mounted with coverslips and prepared for microscopic examination. The intensity of immunohistochemical and fluorescence signals was quantified digitally using ImageJ analysis software (National Institutes of Health).

**TABLE 2:** Antibody information for IHC/IF.

Antibody	Catalogue	Brand	Origin	Concentration	Application
ALP	ET1601‐21	Huabio	Zhejiang, China	1:200	IHC
OCN	A20800	ABclonal	Hebei, China	1:200	IHC
NOX2	HA723224	Huabio	Zhejiang, China	1:200	IHC
RUNX2	ET1612‐47	Huabio	Zhejiang, China	1:200	IHC
CD90	66,766–1‐Ig	Proteintech	Hubei, China	1:500	IF
COL1A1	67,288–1‐Ig	Proteintech	Hubei, China	1:500	IF
Anti‐rabbit IgG (H + L)	8889S	CST	Massachusetts, Aerican	1:1000	IF
Anti‐mouse IgG (H + L)	4408S	CST	Massachusetts, Aerican	1:1000	IF

### 2.7 Dihydroethidium (DHE) Staining

The In vivo assessment of ROS levels within the tissue was conducted through DHE fluorescence staining based on a standardized protocol from a kit (BestBio, BB‐470516, China). Tissue samples were successively treated with dewaxing and rehydration, followed by a five‐minute soak in a clearing agent. Subsequently, freshly prepared DHE staining solution was evenly applied to the sections, which were kept in the dark for 30 min. Post‐staining, samples received three PBS washes before DAPI counterstaining (10 min). Following final mounting, fluorescent signals from both DHE and DAPI channels were captured using a Zeiss fluorescence microscope (Germany). Quantitative analysis of positively stained regions was performed through ImageJ software processing.

### 2.8 Preparation of Drug Serum

The preparation of medicated serum was based on methods from our previous research [24]. Twenty male Sprague–Dawley rats (12‐week‐old, 200 ± 20 g body weight) were allocated randomly into two cohorts. The treatment group received daily oral administration of YGPs (2.3 g/kg) for seven consecutive days, while control animals were administered equivalent volumes of normal saline. Post‐euthanasia cardiac puncture was performed for blood collection. Following centrifugation at 3000 rpm for 10 min, serum fractions were separated through sequential filtration and heat‐inactivation processes (56°C for 30 min) before cryopreservation at −80°C. The drug serum was also analysed via UPLC‐Q‐TO F/MS.

### 2.9 Cell Culture

The MSC C3H10T1/2 lineage (ATCC, USA) was maintained in α‐MEM medium (Gibco, USA) supplemented with 10% FBS (Gibco, USA) and 1% penicillin–streptomycin solution (Gibco, USA). Cell cultures were sustained under standard incubation conditions (37°C, 5% CO_2_ humidified atmosphere) with regular medium replacement every 48 h.

### 2.10 Cell Viability Analysis

Cells were plated in 96‐well plates at an initial density of 5 × 10^3^ cells/well and incubated for 24 h, 48 h, and 72 h with culture medium containing varying proportions (0%, 2.5%, 5%, 7.5%, 10%, 12.5%, and 15%) of YGPs‐containing rat serum. Subsequently, the cells were exposed to H_2_O_2_ (Sigma, 7722‐84‐1, USA) for 24 h. H_2_O_2_ concentrations refer to previous studies [25]. After 24 h, cell viability was detected using a microplate reader (Biotek Synergy, USA), and the specific steps were carried out in accordance with the CCK‐8 reagent (BIOSS, BA00208, China) instructions.

### 2.11 EdU Staining

Cell proliferation of MSCs was assessed using the EdU fluorescence labeling kit (Beyotime, Cat. No. C0081S). The experimental protocol involved plating MSCs in 24‐well culture plates at 5 × 10^4^ cells/well density and allowing them to adhere overnight. Following cell attachment, experimental groups were established and treated according to designated protocols. Post‐treatment, cells were exposed to 25 μM EdU‐containing medium for 2 h before PBS washing. Subsequent processing included 15‐min fixation with 4% paraformaldehyde, PBS rinsing, and 10‐min membrane permeabilization using 0.5% Triton X‐100. After additional PBS washes, cells underwent 30‐min dark incubation with fluorescent azide/Cu^2+^ click reaction solution. Nuclear counterstaining was performed with DAPI for 5 min followed by final PBS rinses. Fluorescence microscopy imaging was conducted, with quantitative analysis executed through ImageJ software.

### 2.12 TUNEL Staining

Following treatment, cellular specimens underwent fixation with 4% paraformaldehyde and subsequent PBS rinsing. Cells were then treated with TUNEL reaction mixture under light‐protected conditions for 1 h. After thorough washing, nuclear counterstaining was performed using DAPI solution for 10 min followed by final PBS washes. Fluorescent signals were documented through microscopic imaging and quantified using ImageJ analysis software.

### 2.13 DCFH‐DA Staining

The accumulation of ROS in MSCs was evaluated using the DCFH‐DA fluorescence probe method (Beyotime, Cat. No. S0033S). Briefly, MSCs were seeded in culture plates to adhere. After the designated treatment intervention, the cells were incubated with DCFH‐DA working solution in the dark for 30 min to allow the probe to be taken up by the cells and then hydrolyzed by intracellular esterases to DCFH. Post‐incubation, three PBS washes were performed to remove residual probe. Nuclear counterstaining was performed using Hoechst solution for 10 min prior to final rinsing. Fluorescence microscopy imaging was conducted to visualize intracellular signals, with quantitative analysis of fluorescence intensity performed through ImageJ software.

### 2.14 Nile Red Staining

Lipid accumulation in MSCs was detected by Nile Red fluorescence staining (Beyotime, Cat. No. C2051S). The specific steps were as follows: After the designated treatment intervention, cells were fixed with paraformaldehyde, washed with PBS, and then incubated with Nile Red working solution for 30 min. After the incubation, cells were thoroughly washed, stained with DAPI for 10 min, and washed for the last time. Fluorescent signals were documented through microscopic imaging and quantified using ImageJ analysis software.

### 2.15 C11 BODIPY Staining

The degree of lipid peroxidation in MSCs was detected by C11 BODIPY fluorescence staining (Beyotime, Cat. No. S0043S). The specific steps were as follows: After the designated treatment intervention, the cells were washed and then incubated with freshly prepared C11 BODIPY working solution for 30 min. After the incubation, the cells were thoroughly washed, stained with Hoechst for 10 min, and washed for the last time. Fluorescent signals were documented through microscopic imaging and quantified using ImageJ analysis software.

### 2.16 Cellular Osteogenic Differentiation and Mineralization

The cells were digested and resuspended to 50,000 cells/ml, and inoculated into a 24‐well plate. The cells were then cultured for osteogenic induction and divided into intervention groups. The induction medium was prepared according to a previous study. Following a week‐long induction period, the cells were collected, fixed, washed, and then stained with an ALP staining kit to detect ALP activity (Thermo Fisher Scientific, 34,042, USA). After a three‐week induction period, the cells were collected, fixed, washed, and then stained with an ARS staining solution (Beyotime, C0148S, China).

### 2.17 Wound Healing Assay

The migration ability of MSCs was evaluated through a wound healing assay. The experimental protocol included the following steps: MSCs suspension was seeded at a density and cultured in a 6‐well plate under standard conditions (37°C, 5% CO_2_) until the cell confluence reached 90%–100%. After a monolayer was formed, a uniform linear wound (approximately 1 mm wide) was created on the culture surface in a vertical direction using a sterile 200 μL pipette tip. Cellular debris elimination involved triple‐wash procedures with phosphate‐buffered saline before introducing drug‐supplemented culture medium. The progress of wound closure was continuously recorded at baseline (0 h) and 24‐h intervals using a phase contrast microscope. Subsequently, the wound area was quantified using ImageJ analysis software to compare the cell migration rate.

### 2.18 Transfection of siRNA‐Nrf2

To knock down Nrf2 expression, siRNA transfection was performed using X‐tremeGENE siRNA Transfection Reagent (Roche, XTG9‐RO, Basel, Switzerland) with either Nrf2‐targeting siRNA or scrambled control siRNA (Jima Pharmaceutical, Shanghai, China). Actively dividing cells were plated at optimal density and incubated with freshly prepared siRNA complexes in serum‐deprived medium over a 6–8 h period. Following transfection, cultures were maintained in complete growth medium supplemented with experimental compounds as required.

### 2.19 Western Blot Analysis

Cells were digested, resuspended to 50,000 cells/mL, and seeded into 24‐well plates. After cell treatment, the cell protein was collected, the concentration was quantified and a subsequent Western blot experiment was performed. For corresponding antibody information, refer to Table 3. For a detailed description of the experimental procedures, please refer to our previous studies.

**TABLE 3:** Antibody information for Western blot.

Antibody name	Company brand	Item number	Dilution ratio
GAPDH	Huabio	ET1601‐4	1:5000
β‐Actin	Proteintech	20,536–1‐AP	1:5000
Osterix	Hua Bio	ER1914‐47	1:1000
Runx2	Hua Bio	ET1612‐47	1:1000
OCN	ABclonal	A20800	1:1000
Nrf2	Immunoway	YT3189	1:1000
HO‐1	Huabio	ER1802‐73	1:1000
IgG (H + L)	Proteintech	SA00001‐2	1:10,000

### 2.20 Real‐Time Quantitative Real Time‐Polymerase Chain Reaction (qPCR) Analysis

Cells were digested, resuspended to 50,000 cells/ml, and seeded in a 12‐well plate. The pre‐treatment intervention lasted 24 h, followed by a 24‐h H_2_O_2_ treatment. Post‐treatment, RNA extraction from cells and subsequent PCR detection were carried out according to our previous experimental methods. The primers were synthesized by Sangon Bio (Shanghai, China), with the forward and reverse sequences of the target genes detailed in Table 4.

**TABLE 4:** Primer name and sequences for qPCR analysis.

Gene	Forward sequence	Reverse sequence
*β‐Actin*	5′‐CATTGCTGACAGGATGCAGAAGG‐3′	5′‐TGCTGGAAGGTGGACAGTGAGG‐3′
*Osterix*	5′‐GGCTTTTCTGCGGCAAGAGGTT‐3′	5′‐CGCTGATGTTTGCTCAAGTGGTC‐3′
*Runx2*	5′‐CCTGAACTCTGCACCAAGTCCT‐3′	5′‐TCATCTGGCTCAGATAGGAGGG‐3′
*OCN*	5′‐GCAATAAGGTAGTGAACAGACTCC‐3′	5′‐CCATAGATGCGTTTGTAGGCGG‐3′
*COL1A1*	5′‐CCTCAGGGTATTGCTGGACAAC‐3′	5′‐CAGAAGGACCTTGTTTGCCAGG‐3′
*ALP*	5′‐CCAGAAAGACACCTTGACTGTGG‐3′	5′‐TCTTGTCCGTGTCGCTCACCAT‐3′
*Nrf2*	5′‐CAGCATAGAGCAGGACATGGAG‐3′	5′‐GAACAGCGGTAGTATCAGCCAG‐3′
*HO‐1*	5′‐CACTCTGGAGATGACACCTGAG‐3′	5′‐GTGTTCCTCTGTCAGCATCACC‐3′

### 2.21 Analysis of GEO Dataset

The transcriptomic datasets analysed in this investigation were sourced from the Gene Expression Omnibus (GEO) repository maintained by the National Center for Biotechnology Information (https://www.ncbi.nlm.nih.gov/geo/). The data processing procedure is as follows: First, GSE datasets of osteoporosis patient samples were sought in the GEO database, and then GSE datasets of bone tissue samples or human MSCs samples were selected. Differential gene analysis was conducted using the online platform easyGEO (https://tau.cmmt.ubc.ca/eVITTA/easyGEO/), with adjusted thresholds of *p* < 0.05 and |fold change| > 1.5 for identifying differentially expressed genes. Functional enrichment assessments for Gene Ontology categories and KEGG pathways were subsequently executed utilizing the cluster Profiler package within the R programming framework. All analyses were conducted in the R 4.0.3 environment, with a significance threshold set at *p* < 0.05.

### 2.22 Network Pharmacology Analysis

The bioactive constituents and molecular targets of YGPs were obtained from the Traditional Chinese Medicine Systems Pharmacology Database (TCMSP) [26]. The screening parameters of the TCMSP database were oral bioavailability ≥ 30% and drug‐likeness score ≥ 0.18 for compounds. For compounds satisfying these parameters but lacking target annotations, structural data from PubChem facilitated target prediction through the Swiss Target Prediction platform. Then, the UniProt database was used to match the predicted protein targets with their corresponding gene names to standardize the protein target information and obtain gene symbols. Disease‐related target genes were determined by searching for “osteoporosis” in multiple online databases, including Genecards, OMIM, TTD, and Drugbank. The cross‐targets of drugs and diseases were achieved through the Venn method. These cross‐targets were imported into the STRING database for analysis to obtain protein–protein interaction (PPI) information, and the PPI relationship map was explored using the STRING database. Enrichment analysis was conducted through online platforms (https://www.bioinformatics.com.cn/), and the GO and KEGG methods were used to reveal key biological processes and signalling pathways [27].

### 2.23 Molecular Docking

Molecular docking was performed using AutoDock Vina (http://vina.scripps.edu/). The crystal structure of the target protein was retrieved from the Protein Data Bank (PDB, https://www.rcsb.org/). Both the protein and ligand were prepared in accordance with the standard docking protocol, and docking simulation was conducted at the predicted active site. Binding affinity was evaluated via the docking energy score: a higher absolute value indicates a stronger predicted interaction between the protein and ligand. Representative docking conformations were further analysed to identify key interacting residues and spatial complementarity.

### 2.24 Statistical Analysis

GraphPad Prism 8.0 (GraphPad, CA, USA) was used for statistical analysis. All descriptions of the experimental data were shown as the mean ± standard deviation (SD). One‐way ANOVA and Dunnett's *t*‐test were used to compare groups. *p* < 0.05 was regarded as statistical significance.

## 3 Results

### 3.1 Identification of Active Components in YGPs and YGPs‐Containing Drug Serum

**FIGURE 1:**
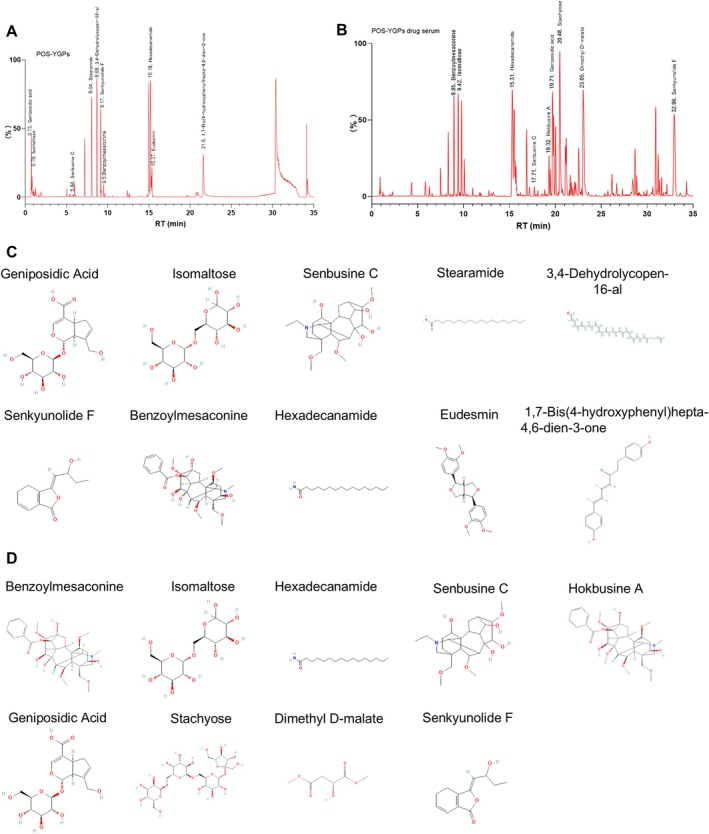
Identification of bioactive Compounds in YGPs and YGPs‐containing sera by ultra‐performance Liquid Chromatography (UPLC). (A) UPLC chromatograms of YGPs in positive ion mode, highlighting the 10 most representative compounds. (B) Chemical structure of key bioactive components in YGPs. (C) UPLC chromatogram of YGPs‐containing sera in positive ion mode, highlighting the nine most representative compounds. (D) Chemical structure of key bioactive components of YGPs‐containing serum.

To identify the bioactive components of YGPs and YGPs‐containing serum and explore its potential pharmacological relevance, the samples were subjected to UPLC‐Q‐TOF/MS analysis (Figure 1A,B). Through the comparison of retention times, response values, and published literature with reference standards, 10 key compounds in YGPs were preliminarily identified (Figure 1C; Table S1). Similarly, 9 key compounds in YGPs‐containing serum were identified (Figure 1D; Table S2). Among these, Geniposidic Acid, Isomaltose, Senbusine C, Senkyunolide F, Benzoylmesaconine, and Hexadecanamide were abundantly present in the serum, representing the primary systemic exposure components.

### 3.2 YGPs Significantly Delayed Bone Mass Loss in OVX Mice

**FIGURE 2:**
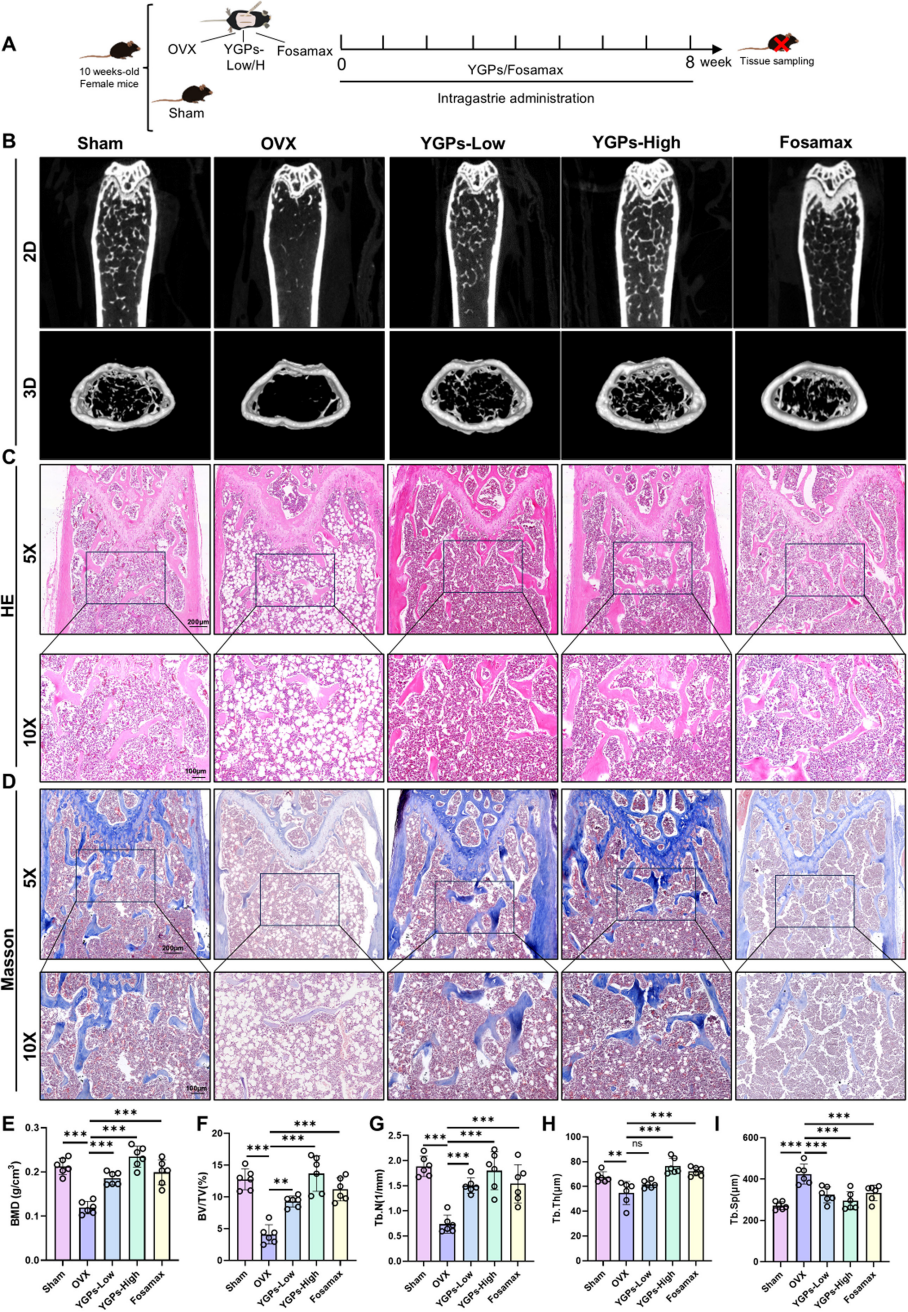
Therapeutic Effect of YGPs on bone mass and bone microstructure in ovariectomized osteoporotic mice. (A) Schematic diagram of the experimental design. (B) Representative 2D and 3D micro‐CT images of trabecular bone in distal femurs of mice from different treatment groups, showing trabecular bone architecture changes in different groups. (C) H&E staining at different magnifications showed restoration of trabecular bone structure. (D) The coverage of collagen fibres in each group was visualized by Masson staining at different magnifications. (E–I) Statistical data of BMD, BV/TV, Tb.N, Tb.Th, and Tb.Sp were collected in each group. Experimental groups (Sham, OVX, YGPs‐Low, YGP‐High, Fosamax). All data are presented as mean ± SD. Statistical significance was expressed as *p < 0.05, **p < 0.01, and ***p < 0.001 (*n* = 6).

Building on the chemical characterization, we next evaluated the In vivo therapeutic efficacy of YGPs using an ovariectomy‐induced osteoporosis mouse model (Figure 2A). Micro‐CT reconstructions (Figure 2B) revealed severe trabecular loss in femoral metaphysis among model group specimens compared with sham controls, while YGPs‐treated groups exhibited trabecular network regeneration. H&E staining and Masson staining further demonstrated that the trabecular area in the YGPs treatment groups was remarkably restored, and the number of fat vacuoles was dramatically reduced compared to the model group (Figure 2C,D). Quantitative CT analysis (Figure 2E–H) showed that BMD, BV/TV, Tb.N, and Tb.Th were markedly lower in the model group relative to sham controls. Treatment with YGPs dose‐dependently improved these bone microstructure parameters. Among them, the high‐dose YGPs group exhibited the most robust restorative effect, which was comparable to or even superior to that of the positive control drug, Fosamax. Additionally, Tb.Sp in the model group was significantly increased but was normalized to near‐sham levels after high‐dose YGPs treatment (Figure 2I).

### 3.3 YGPs Significantly Improved the Bone Formation Capacity of OVX Mice

**FIGURE 3:**
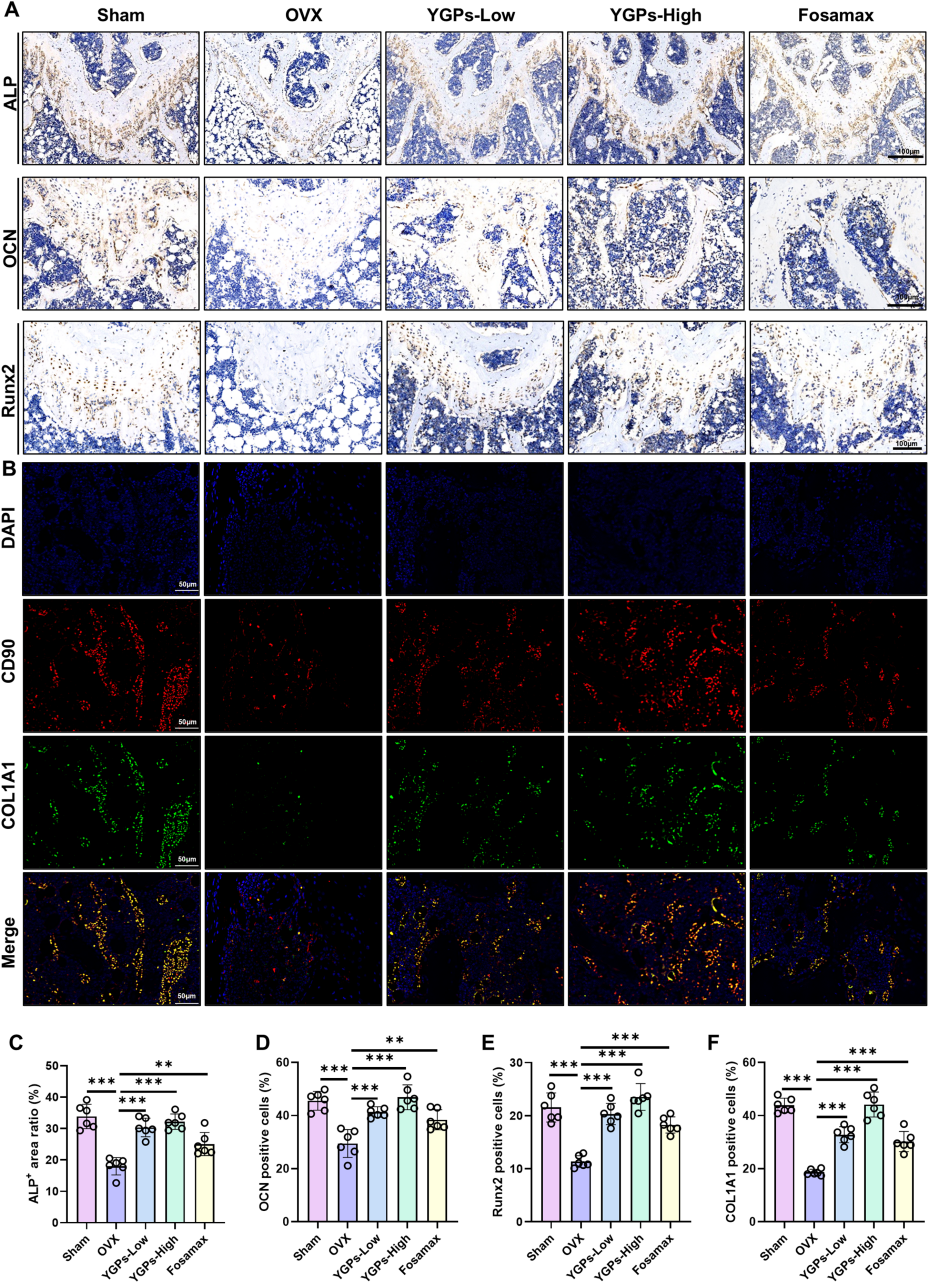
YGPs promotes bone formation in osteoporosis. (A) Positive expression of ALP, OCN and RUNX2 near the femoral growth plate in each group. (B) The expression level of COL1A1 in CD90‐labelled mesenchymal stem cells (MSCs) isolated from femurs in each group is presented, where DAPI stains the cell nuclei blue, CD90 labeling is stained red, COL1A1 labeling is stained green, and the merged image presents an orange colour. (C–F) Statistical analysis of ALP, OCN, RUNX2 and COL1A1 positive expression in each group All data are presented as mean ± SD. Statistical significance was expressed as *p < 0.05, **p < 0.01 and ***p < 0.001 (*n* = 6).

Given the observed improvements in bone microstructure, we further investigated whether YGPs promote osteogenic activity In vivo using immunohistochemical staining. Compared with the sham control group, the model group exhibited a marked reduction in the ALP positive area and in the number of OCN and Runx2 positive cells. In contrast, the YGPs group showed elevated expression levels of ALP, OCN, and Runx2 compared to the model group (Figure 3A,C–E). Since MSCs serve as the progenitor cells for osteoblasts, we next assessed whether YGPs modulate MSC osteogenic differentiation In vivo. The results demonstrated that YGPs enhanced the expression of Runx2 in MSCs, with the high‐dose YGPs group exhibiting higher Runx2 expression than the Fosamax group (Figure 3B,F). These findings indicate that YGPs promote bone formation, at least in part, by enhancing MSC osteogenic commitment.

### 3.4 The Therapeutic Effect of YGPs on OVX Mice Is Associated With the Reduction of ROS Accumulation

To elucidate the molecular mechanisms underlying YGPs' osteoprotective effects, we first performed bioinformatic analysis of publicly available osteoporosis datasets. Analysis of the GSE35956 dataset identified 972 differentially expressed genes in the femurs of Non‐OP and OP patients, among which 459 genes were upregulated and 513 genes were downregulated (Figure S1A,B). GO and KEGG enrichment analyses revealed dysregulation of redox homeostasis as a top altered biological process (Figure S1C–F).

**FIGURE 4:**
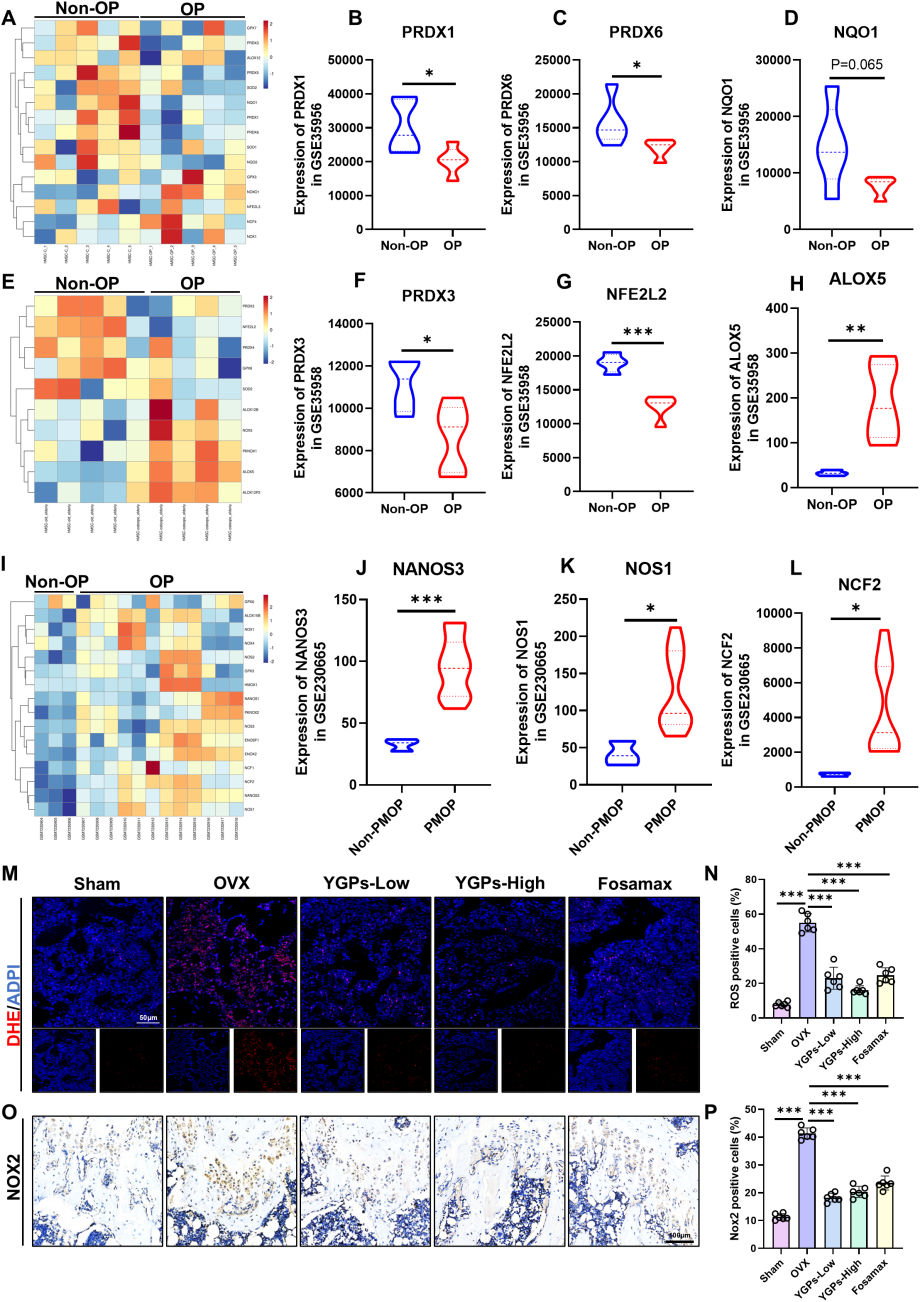
Progression of osteoporosis is closely related to ROS accumulation, which is inhibited by YGPs in osteoporotic mice. (A) Heat map analysis of oxidative stress‐related gene expression in GSE35956. (B–D) Expression of oxidative stress‐related genes (PRDX1, PRDX6, NQO1) in GSE35956. (E) Heat map analysis of oxidative stress‐related gene expression in GSE35958. (F–H) Expression of oxidative stress‐related genes (PRDX3, NFE2L2, ALOX5) in GSE35958. (I) Heat map analysis of oxidative stress‐related gene expression in GSE230665. (J–L) Expression of oxidative stress‐related genes (NANOS3, NOS1, NCF2) in GSE230665. (M, N) Typical DHE‐stained images and quantitative analysis showed a significant increase in ROS in osteoporotic mice and a reduction in ROS accumulation in osteoporotic femoral bone sections treated with YGPs (*n* = 6). (O, P) Typical NOX2 histochemical staining images and quantitative analysis showed that the expression of NOX2 in the femurs of osteoporotic mice treated with YGPs was significantly reduced compared with the model group (*n* = 6). All data are presented as mean ± SD. Statistical significance was expressed as *p < 0.05, **p < 0.01, and ***p < 0.001, respectively.

Consistent with this finding, focused analysis of oxidative stress‐related genes in the GSE35956 dataset (Figure 4A) showed that antioxidant genes (including PRDX1, PRDX6, NQO1) were downregulated (Figure 4B–D). A similar expression pattern was also observed in the GSE35958 dataset. We performed differentially expressed gene analysis, GO analysis and KEGG analysis on this dataset (Figure S2A–F). The results showed that the expression of antioxidant genes, including PRDX3 and NFE2L2, was downregulated, while the expression of ALOX5, a gene that promotes ROS production, was upregulated (Figure 4E–H). Analysis of the GSE230665 dataset further validated this finding. We conducted differentially expressed gene analysis, GO analysis and KEGG analysis on this dataset (Figure S3A–F), and confirmed that in patients with PMOP, the expression levels of genes that promote ROS accumulation, including NANOS3, NOS1 and NCF2, were elevated (Figure 4I–L).

To validate these bioinformatic findings, we measured oxidative stress markers in bone tissues from OVX mice. Results indicated a marked elevation in ROS levels within the model group, while YGPs‐treated cohorts demonstrated notably reduced ROS concentrations compared to untreated counterparts (Figure 4M,N). Quantitative analysis further showed substantial upregulation of NOX2 expression in pathological models, which was effectively counteracted through YGPs administration (Figure 4O,P).

### 3.5 YGPs Drug‐Containing Serum Effectively Reduced the Oxidative Stress Injury and Apoptosis of MSCs Induced by H_2_O_2_

**FIGURE 5:**
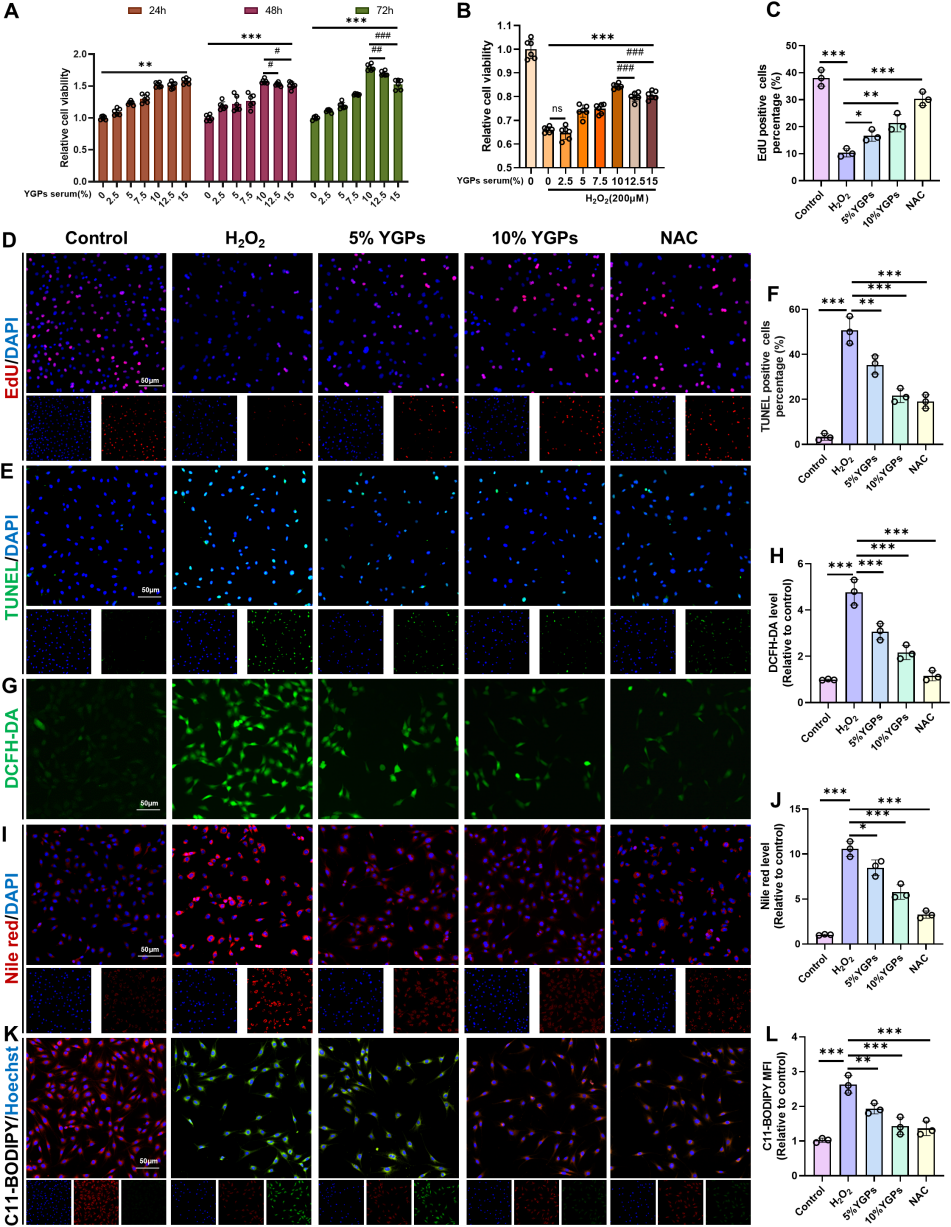
YGPs‐containing serum effectively reduced H_2_O_2_‐induced oxidative stress injury and apoptosis of MSCs. (A) The effect of different concentrations of YGPs‐containing serum administration on the viability of MSCs was detected by CCK‐8 (*n* = 6). (B) Cell viability of H_2_O_2_‐treated MSCs after administration of YGPs‐containing serum was determined by CCK‐8 (*n* = 6). (C, D) Representative images and quantitative analysis of EdU staining (*n* = 3). (E, F) Representative images of TUNEL staining and quantitative analysis (*n* = 3). (G, H) Representative images of DCFH‐DA staining and quantitative analysis (*n* = 3). (I, J) Representative images and quantitative analysis of Nile red staining (*n* = 3). (K, L) Representative images and quantitative analysis of C11 BODIPY staining (*n* = 3). All data are presented as mean ± SD. Statistical significance was expressed as *p < 0.05, **p < 0.01, and ***p < 0.001.

To establish a direct causal link between YGPs, oxidative stress, and MSC function, we next employed an In vitro H_2_O_2_‐induced oxidative stress model. Firstly, CCK‐8 was used to detect the cell viability after 24, 48, and 72 h of intervention with YGPs drug‐containing serum. The results showed that YGPs drug‐containing serum promoted the viability of MSCs, suggesting that it promoted the proliferation of MSCs, and the optimal effect was achieved at a concentration of 10% (Figure 5A). Additionally, under the H_2_O_2_‐induced oxidative damage model, YGPs drug‐containing serum dose‐dependently restored the viability of MSCs, and the optimal effect was achieved at a concentration of 10% (Figure 5B).

Moreover, EdU fluorescence staining also indicated that YGPs drug‐containing serum improved the damage to the proliferation ability of MSCs caused by H_2_O_2_ (Figure 5C,D). Meanwhile, TUNEL results showed that H_2_O_2_‐induced oxidative damage to MSCs led to a significant increase in cell apoptosis, while the intervention of YGPs drug‐containing serum effectively reduced cell apoptosis (Figure 5E,F). DCFH‐DA fluorescence staining demonstrated that H_2_O_2_ treatment caused robust ROS accumulation, which was significantly blunted by YGPs‐containing serum (Figure 5G,H).

Beyond total ROS, we also examined lipid peroxidation, a key downstream consequence of oxidative stress. Nile Red fluorescence staining showed that H_2_O_2_ treatment caused the accumulation of lipids, and the intervention of YGPs drug‐containing serum reduced the accumulation of lipids (Figure 5I,J). C11 BODIPY 581/591 fluorescence staining further confirmed that H_2_O_2_ treatment induced marked lipid ROS accumulation, which was effectively suppressed by YGPs intervention (Figure 5K,L).

### 3.6 The Drug‐Containing Serum of YGPs Effectively Enhanced the Osteogenic Differentiation and Migration Ability of MSCs

**FIGURE 6:**
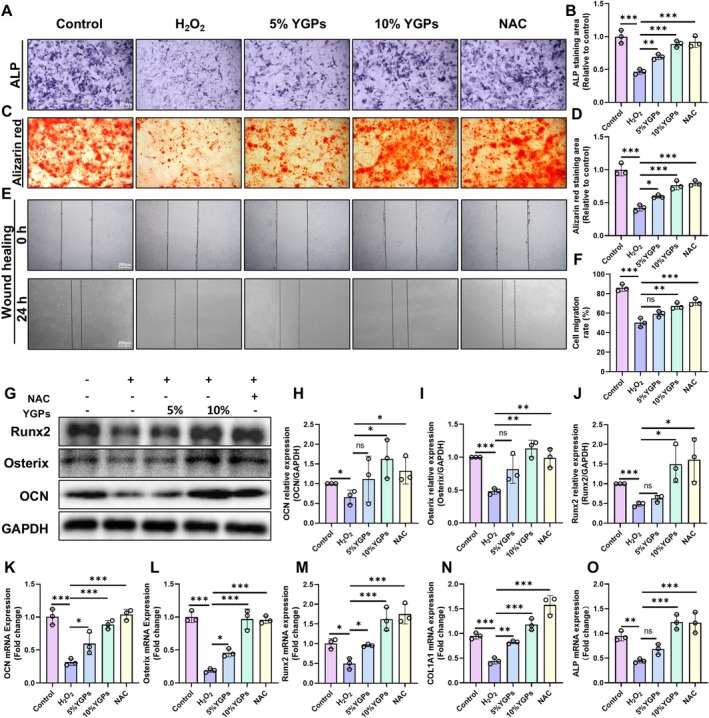
YGPs‐containing serum effectively enhanced osteogenic differentiation and migration of MSCs. (A, B) Representative images and quantitative analysis of ALP staining. (C, D) Representative images and quantitative analysis of alizarin red staining. (E, F) Representative images and quantitative analysis of cell scratch assays. (G–J) Western blot images and analysis confirmed that treatment with YGPs‐containing serum upregulated the protein expression of RUNX2, Osterix, and OCN in MSCs. (K–O) qPCR results showed that treatment with YGPs‐containing serum upregulated the mRNA expression of OCN, Osterix, Runx2, COL1A1, and ALP in MSCs. All data are presented as mean ± SD. Statistical significance was expressed as *p < 0.05, **p < 0.01, and ***p < 0.001, respectively (*n* = 3).

Having established that YGPs protect MSCs from oxidative damage, we next investigated whether this protection translates to preserved cellular function. ALP staining results showed that the oxidative damage of MSCs caused by H_2_O_2_ led to the impaired synthesis of cellular alkaline phosphatase, while the intervention of YGPs drug‐containing serum effectively enhanced the expression of cellular alkaline phosphatase (Figure 6A,B). Mineralization capacity assessment through alizarin red staining revealed impaired calcium deposition in oxidatively damaged cells, which was substantially improved following YGPs serum treatment (Figure 6C,D). The scratch wound healing assay further showed that YGPs serum intervention effectively counteracted H_2_O_2_‐induced impairment of MSCs' migratory capacity, demonstrating its protective effects on cellular motility. These findings collectively suggest that YGPs serum enhances MSCs' differentiation potential by mitigating oxidative damage‐related functional deficits.

To confirm these observations at the molecular level, we quantified osteogenic differentiation markers. The results showed that H_2_O_2_‐induced oxidative damage in cells inhibited the expression levels of OCN, Osterix, and Runx2 proteins, while YGPs‐containing serum intervention upregulated these osteogenic markers (Figure 6G–J). The qPCR detection results also showed that the mRNA expression of OCN, Osterix, Runx2, COL1A1, and ALP was restored under the intervention of YGPs‐containing drug serum (Figure 6K–O). These results indicated that YGPs‐containing drug serum effectively reduced the oxidative damage of MSCs induced by H_2_O_2_, thereby restoring the osteogenic differentiation and migration functions of MSCs.

### 3.7 The Nrf2 Pathway Is a Key Target for YGPs to Regulate Oxidative Stress in MSCs

**FIGURE 7:**
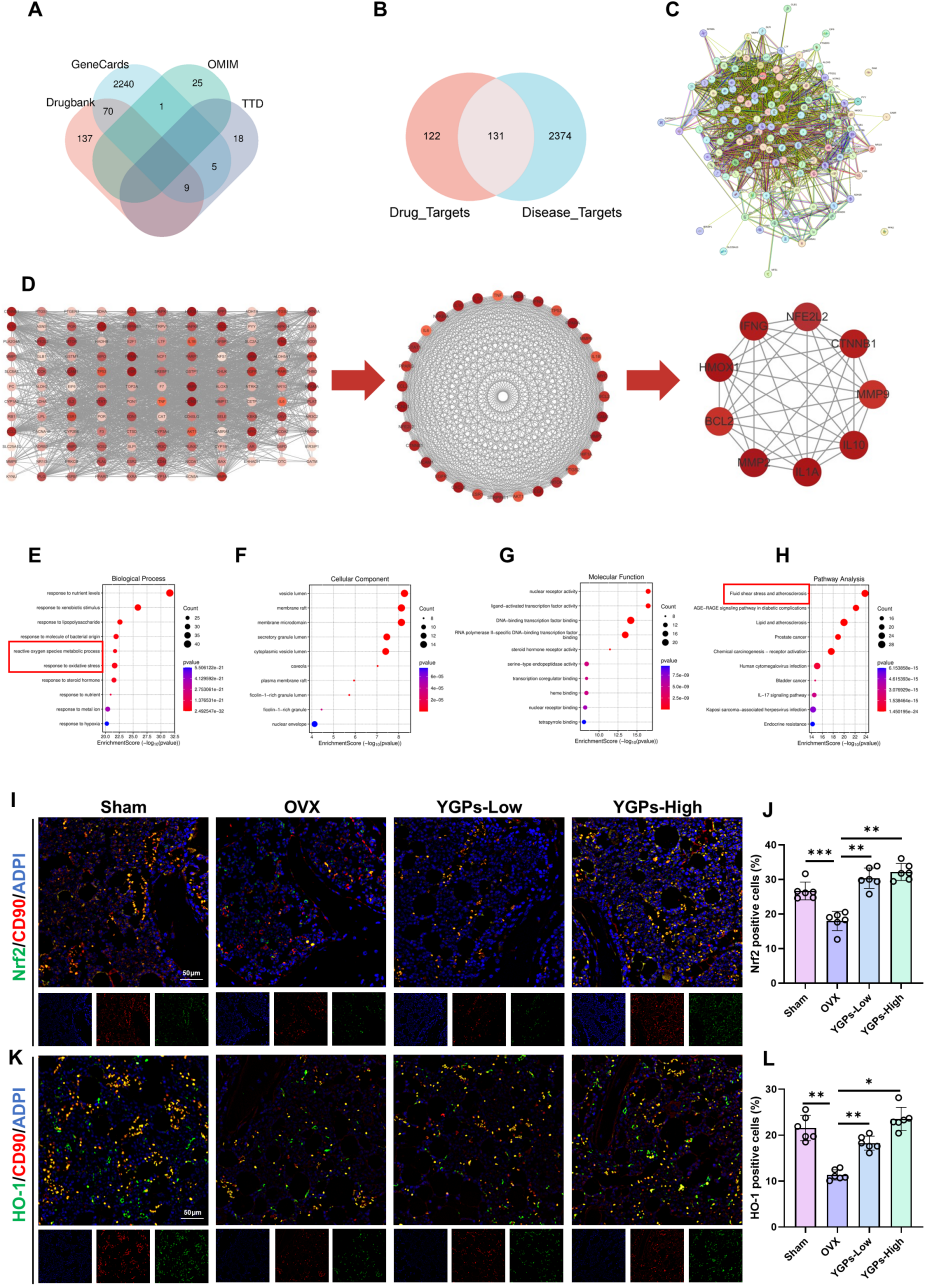
Nrf2 pathway is a key target of YGPs regulating oxidative stress in MSCs from osteoporotic mice. (A) Number of OP disease targets pooled from different database platforms. (B) Venn diagram of intersection of YGPs drugs and OP diseases. (C) PPI network of key targets. (D) Core target network extracted from the PPI network (E–G) Biological Process, Cellular Component and Molecular Function in Go analysis. (H) Pathway Analysis of KEGG. (I–J) Immunofluorescence co‐staining and quantitative analysis of Nrf2 and CD90 in femoral bone marrow sections (*n* = 6). (K, L) Co‐staining and quantitative analysis of HO‐1 and CD90 in femoral bone marrow sections by immunofluorescence (*n* = 6). All data are presented as mean ± SD. Statistical significance was expressed as *p < 0.05, **p < 0.01, and ***p < 0.001, respectively.

To identify the molecular pathway through which YGPs exert their antioxidant effects, we conducted network pharmacology‐based predictive analysis. Osteoporosis was associated with 2505 potential therapeutic targets, while the 10 herbal components of YGPs collectively encompassed 253 targets (Figure 7A). Venn diagram analysis identified 131 overlapping targets potentially mediating YGPs' therapeutic effects against OP (Figure 7B). PPI network analysis revealed central regulatory nodes (Figure 7C,D), and GO and KEGG pathway enrichment analyses implicated the Nrf2 signalling pathway as the predominant mechanistic axis (Figure 7E–H and Figure S4). Then, we conducted molecular docking analyses of major compounds identified—Benzoylmesaconine, Eudesmin, Geniposidic Acid, Hokbusine A, Senbusine C—with the Nrf2‐Keap1 complex. The results showed that Benzoylmesaconine (−8.539 kcal/mol), Eudesmin (−7.597 kcal/mol), Geniposidic Acid (−7.542 kcal/mol), Hokbusine A (−8.548 kcal/mol), and Senbusine C (−8.197 kcal/mol) exhibited significantly higher binding affinities to the Keap1 protein compared to other compounds, enabling them to competitively bind to the Kelch domain of Keap1, thereby releasing Nrf2 and activating downstream signalling pathways (Figure S5).

**FIGURE 8:**
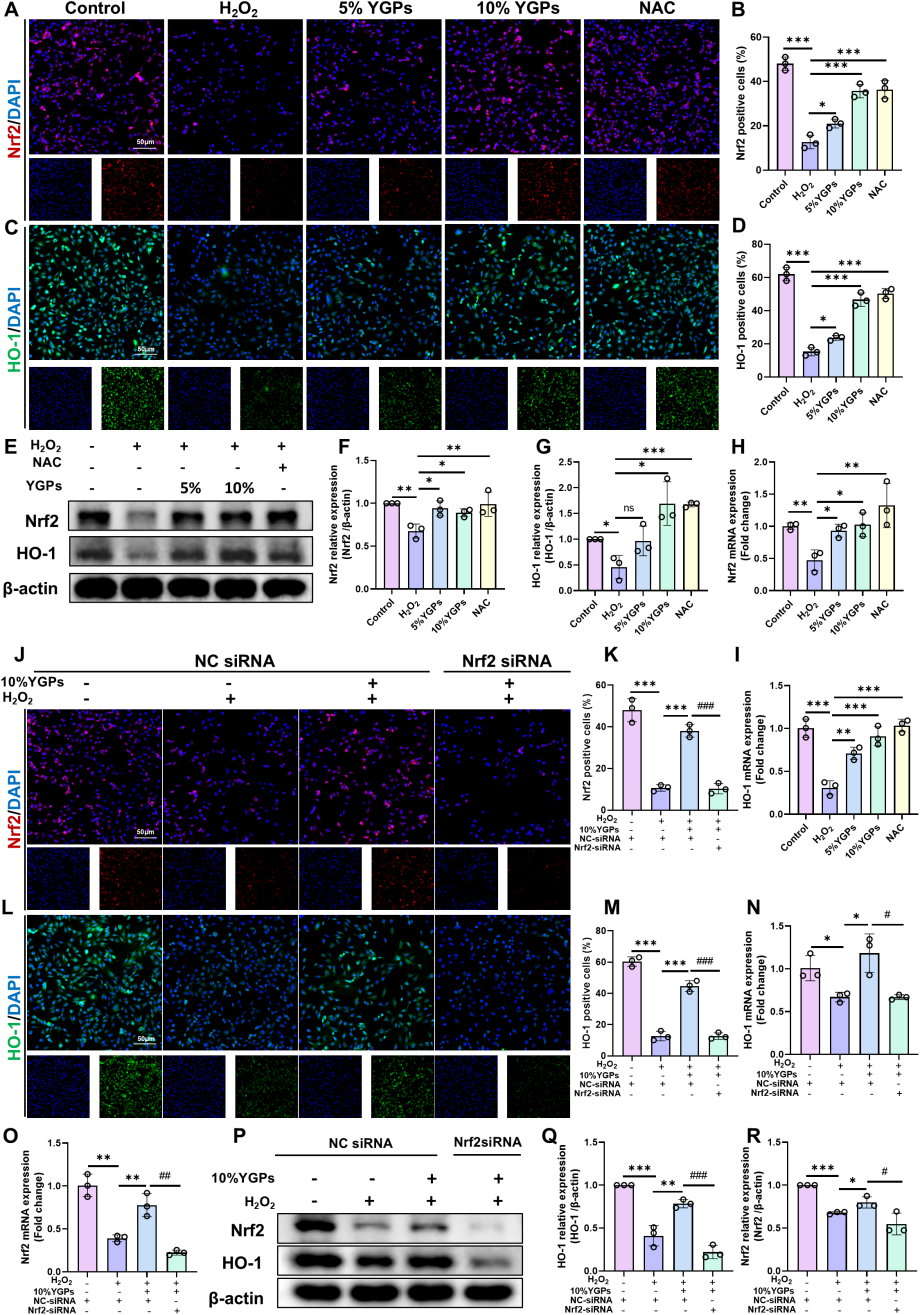
Nrf2 pathway is a key target for YGPs‐containing serum to protect MSCs from H_2_O_2_‐induced oxidative damage. (A, B) Representative images and quantitative analysis of Nrf2 immunofluorescence staining. (C, D) Representative images and quantitative analysis of HO‐1 immunofluorescence staining. (E–G) Western blot images and analysis confirmed that treatment with YGPs‐containing serum upregulated Nrf2 and HO‐1 protein expression in MSCs. (H–I) qPCR results showed that treatment with YGPs‐containing serum upregulated Nrf2 and HO‐1 mRNA expression in MSCs. (J, K) Representative images and quantitative analysis of Nrf2 immunofluorescence staining after si‐Nrf2 interference. (L, M) Representative images and quantitative analysis of HO‐1 immunofluorescence staining after si‐Nrf2 interference. (N, O) qPCR results showed that si‐Nrf2 abolished the effect of YGPs‐containing serum on the expression of Nrf2 and HO‐1 mRNA. (P–R) Western blot analysis demonstrated that si‐Nrf2 abolished the effect of YGPs‐containing serum on Nrf2 and HO‐1 protein expression. All data are presented as mean ± SD. Statistical significance was expressed as *p < 0.05, **p < 0.01, and ***p < 0.001 (*n* = 3).

Consistent with these predictions, validation studies in bone tissue specimens demonstrated that Nrf2 and its downstream effector HO‐1 were downregulated in the OP model group, which was effectively rescued by YGPs administration (Figure 7I–L). Similarly, decreased expressions of Nrf2 and HO‐1 were observed in MSCs treated withH_2_O_2_ In vitro, while YGPs‐containing serum intervention upregulated the expressions of both proteins (Figure 8A–D). Complementary Western blot and qPCR analyses confirmed dose‐dependent upregulation of both mRNA and protein expression for Nrf2 and HO‐1 following YGPs‐containing serum treatment (Figure 8E–I).

To establish causality between Nrf2 activation and YGPs' protective effects, Nrf2‐specific siRNA was employed to disrupt pathway activation. Nrf2 siRNA abolished both YGPs‐mediated Nrf2 activation and its downstream regulatory effects on HO‐1 expression, as evidenced by attenuated mRNA and protein levels of both markers (Figure 8J–R). These findings collectively demonstrate that the Nrf2/HO‐1 axis serves as an essential molecular conduit for YGPs' antioxidative actions in MSCs under oxidative stress conditions.

### 3.8 Knockdown of Nrf2 Eliminated the Antioxidant Protective Effect of YGPs on MSCs

**FIGURE 9:**
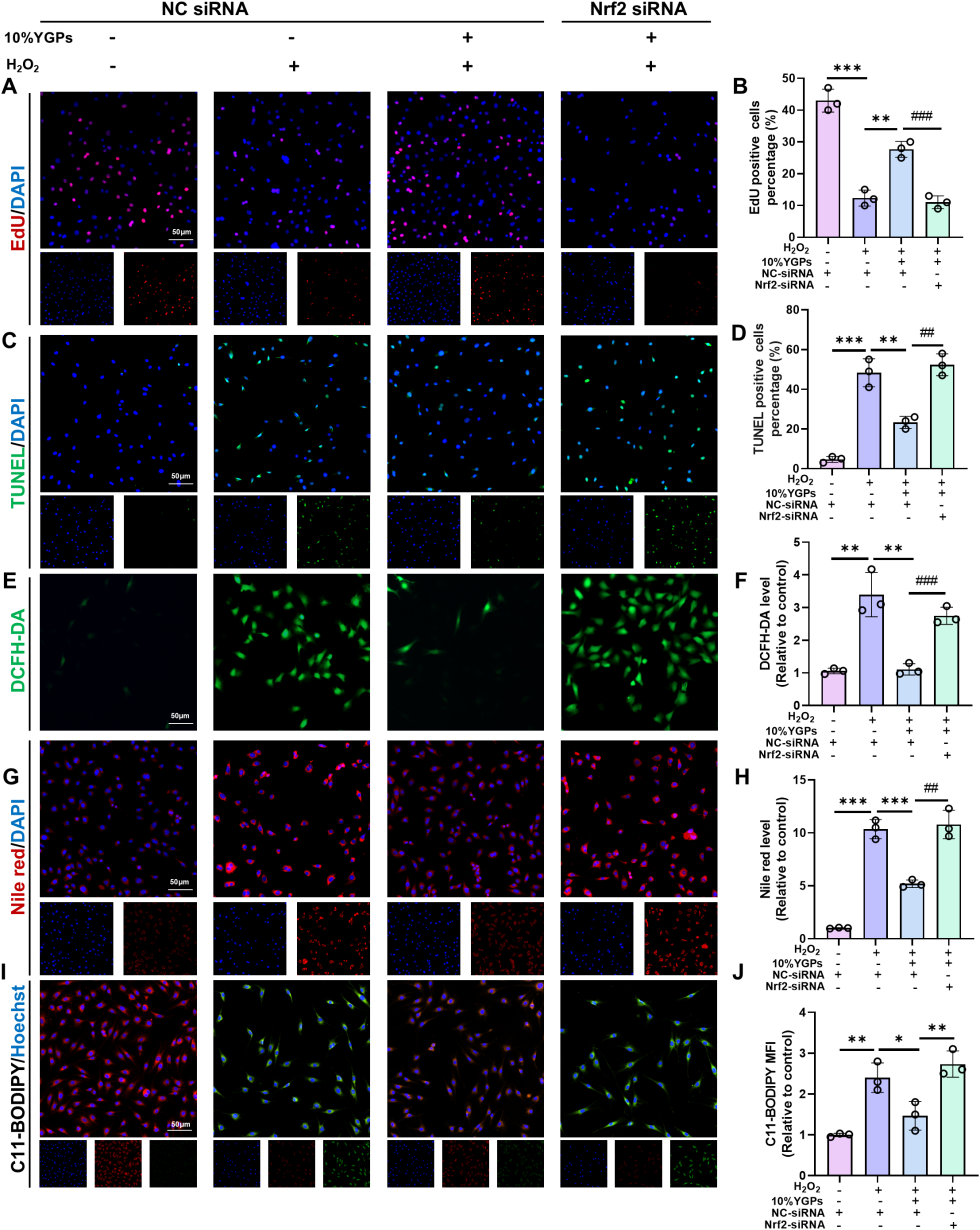
si‐Nrf2 abolished the antioxidant protection of MSCs by YGPs‐containing serum. (A, B) Representative images of EdU staining and quantitative analysis. (C, D) Representative images and quantitative analysis of TUNEL staining. (E, F) Representative images and quantitative analysis of DCFH‐DA staining. (G, H) Representative images and quantitative analysis of Nile red staining. (I, J) Representative images of C11 BODIPY staining and quantitative analysis. All data are presented as mean ± SD. Statistical significance was expressed as *p < 0.05, **p < 0.01, and ***p < 0.001 (*n* = 3).

Having confirmed that YGPs activate the Nrf2 pathway, we next assessed whether this activation is required for YGPs' therapeutic efficacy. CCK‐8 assay revealed no significant difference in the survival rate of MSCs at 24, 48, and 72 h after transfection with Nrf2 siRNA and NC siRNA (Figure S6). The results demonstrated that Nrf2 siRNA transfection abolished the proliferative enhancement of MSCs induced by YGPs (Figure 9A,B). TUNEL assays further revealed that Nrf2 siRNA administration negated the anti‐apoptotic effects of YGPs on MSCs (Figure 9C,D). Fluorescence staining assays (DCFH‐DA, Nile Red, and C11 BODIPY 581/591) confirmed that Nrf2 siRNA treatment eliminated the ROS‐scavenging capacity of YGPs in MSCs (Figure 9E–J).

**FIGURE 10:**
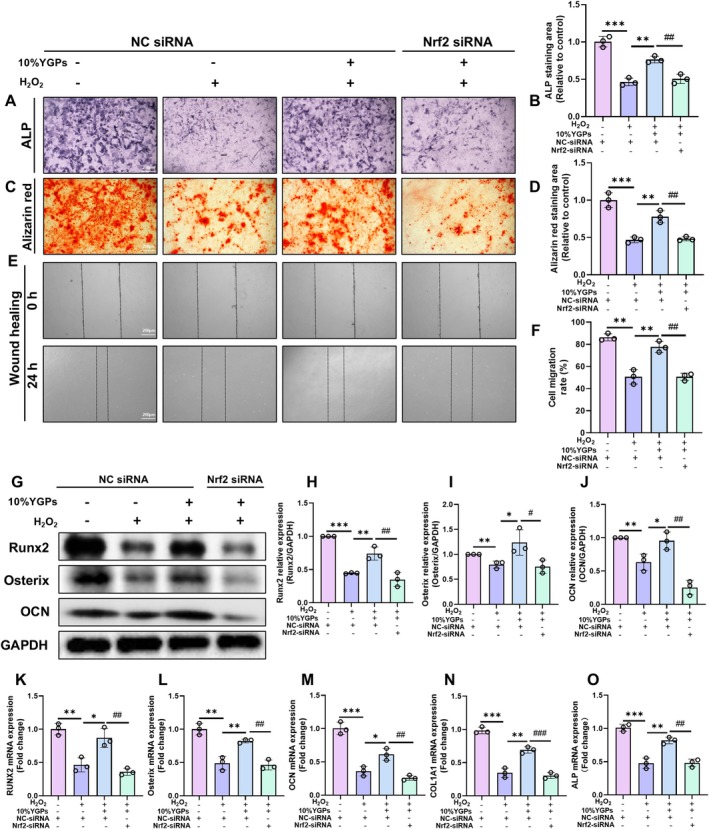
si‐Nrf2 of Nrf2 eliminated the osteogenic differentiation and migration effects of YGPs‐containing drug serum on MSCs. (A, B) Representative images and quantitative analysis of ALP staining. (C, D) Representative images and quantitative analysis of Alizarin Red staining. (E, F) Representative images and quantitative analysis of the cell scratch assay. (G–J) Western blot images and analysis confirmed that si‐Nrf2 eliminated the effects of YGPs‐containing serum on the expression of RUNX2, Osterix, and OCN proteins in MSCs. (K–O) qPCR results indicated that si‐Nrf2 eliminated the effects of YGPs‐containing serum on the mRNA expression of OCN, Osterix, Runx2, COL1A1, and ALP in MSCs. All data are presented as mean ± standard deviation. Statistical significance was indicated by *p < 0.05, **p < 0.01 and ***p < 0.001 (*n* = 3).

Regarding osteogenic differentiation and migratory capabilities, Nrf2 siRNA administration completely abrogated the YGPs‐mediated improvement in MSCs' osteogenic differentiation and migration (Figure 10A–F). Furthermore, Nrf2 knockdown nullified the upregulatory effects of YGPs on protein expression levels of OCN, Osterix, and Runx2, as well as the regulatory effect of YGPs on the mRNA expression of OCN, Osterix, Runx2, COL1A1, and ALP (Figure 10G–O).

These data conclusively establish that the antioxidant protection and functional restoration conferred by YGPs in MSCs are mechanistically dependent on Nrf2 signalling pathway activation.

## 4 Discussion

**FIGURE 11:**
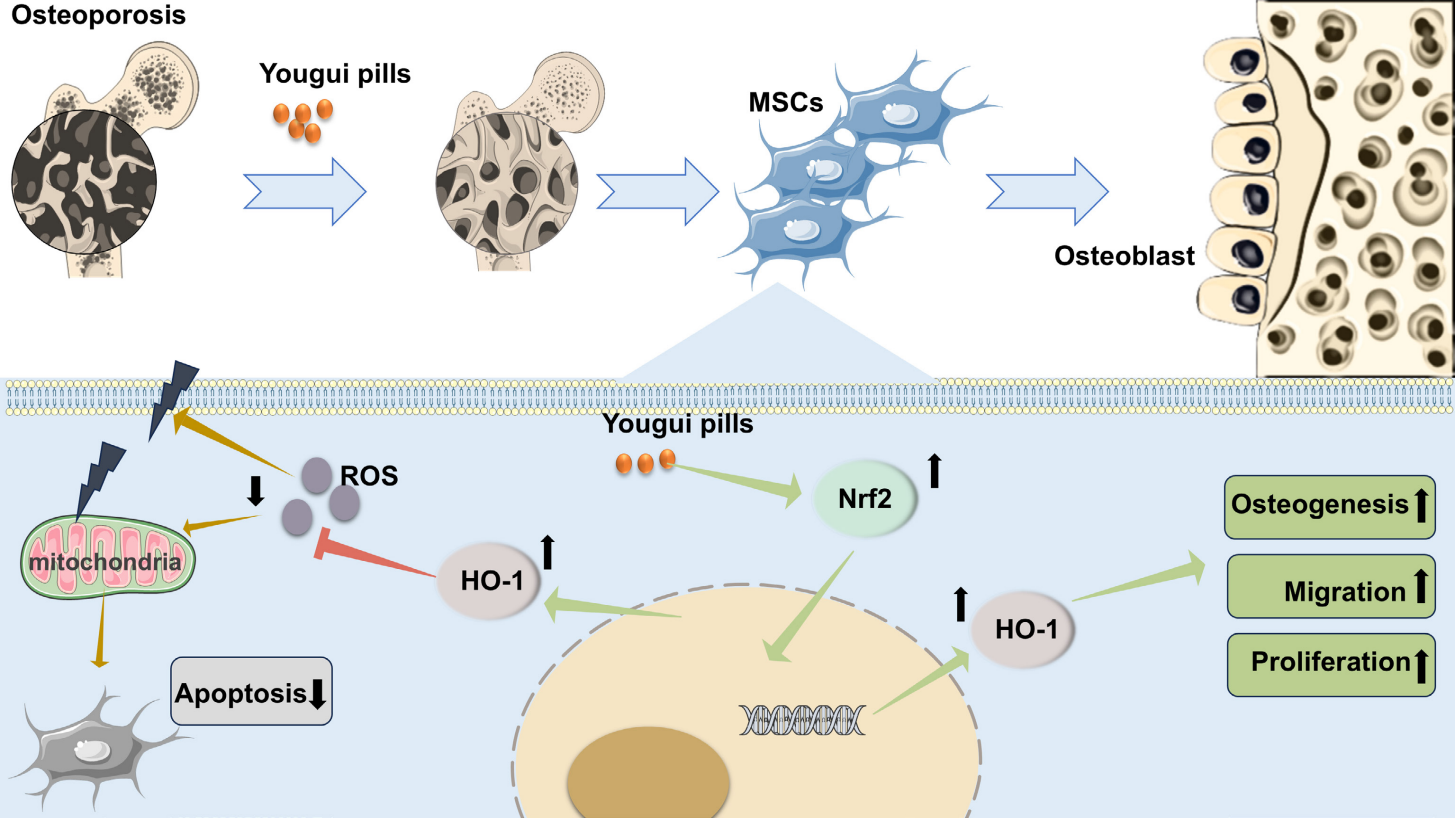
Schematic representation of the mechanism by which Yougui pills inhibits ROS accumulation in MSCs through the Nrf2/HO‐1 pathway to alleviate osteoporosis. YGPs counteract oxidative‐stress‐driven ROS accumulation in MSCs and promote osteogenesis via the Nrf2/HO‐1 pathway, supporting its mechanism‐based potential as a therapeutic option for OP.

Using bioinformatics integrated with In vivo and In vitro experimentation, we show that YGPs exert osteoprotective effects primarily by correcting redox imbalance in the osteoporotic milieu and restoring MSCs' function via activation of the Nrf2/HO‐1 pathway (Figure 11). Functionally, YGPs improved trabecular architecture and bone mass in OVX mice and enhanced osteogenic outputs (ALP, OCN, Runx2, COL1A1). Histological and cell‐based assay results indicated that under oxidative stress conditions, YGPs reduced the levels of total ROS and lipid ROS, decreased apoptosis, restored cell proliferation, migration ability, and reactivated osteogenic differentiation. These findings not only highlight the therapeutic potential of YGPs in treating osteoporosis but also provide mechanistic insights into how traditional Chinese medicine formulas can combat degenerative diseases by modulating key cellular processes.

Oxidative stress—manifested as excessive ROS accumulation—is a central driver of cellular injury in osteoporosis [28, 29]. A sustained redox burden within the bone marrow niche impairs MSCs' proliferation, accelerates apoptosis, and disrupts migration and osteoblast lineage commitment [13, 30, 31, 32]. Re‐analysis of multiple GEO datasets in this study revealed downregulation of antioxidant signatures and upregulation of ROS‐generating pathways in osteoporosis, findings mirrored in OVX mice by elevated ROS and NOX2. In parallel, H_2_O_2_‐exposed MSCs exhibited increased total and lipid ROS, lipid accumulation, diminished proliferation, and suppressed migration and osteogenesis. YGPs mitigated each of these defects, supporting the rationale that limiting ROS accumulation in MSCs is an effective strategy to counter osteoporotic bone loss.

Among the various cellular defence systems against oxidative stress, the Nrf2 pathway stands out as the master regulator of redox homeostasis. Nrf2 orchestrates the activation of antioxidant response elements and detoxification enzymes, thereby protecting cells from oxidative damage [33, 34, 35, 36]. However, in osteoporosis, Nrf2 activity appears compromised, limiting the capacity to clear excess ROS [37, 38]. Notably, experimental models demonstrate that Nrf2 deficiency correlates with skeletal developmental abnormalities and accelerated bone resorption [39, 40, 41]. Consistent with this biological characteristic, we observed that decreased femoral expression of both Nrf2 and its regulatory target HO‐1 in the femur of OVX mice was significantly downregulated, both of which were restored by YGPs. In vitro, YGPs increased Nrf2 and HO‐1 expression in H_2_O_2_‐challenged bone marrow MSCs. Critically, Nrf2 silencing abolished YGPs' antioxidant, antiapoptotic, and pro‐osteogenic benefits, indicating that activation of the Nrf2/HO‐1 axis is necessary for YGPs' MSC‐directed protection.

Phytochemical and serum pharmacochemistry analyses suggest candidate classes that may contribute to Nrf2 engagement. Iridoid glycosides (e.g., geniposidic acid) and phthalide‐type constituents (e.g., senkyunolide F) detected in circulation are reported to possess antioxidant properties and, in some contexts, to modulate Nrf2‐dependent signalling [42, 43, 44, 45]. While the present work was not designed to assign activity to individual molecules, the systemic presence of these constituents supports a plausible multi‐component synergy on redox control and Nrf2 activation. It should be noted that although Nrf2 siRNA in this study almost completely abrogated the antioxidant and osteogenic effects of YGPs, indicating that the Nrf2/HO‐1 axis is the primary pathway through which YGPs exerts its functions, we cannot completely rule out the existence of other compensatory mechanisms.

Our findings reveal that YGPs exert notable anti‐osteoporotic properties in OVX mice through Nrf2/HO‐1 pathway activation, mitigating oxidative stress‐induced impairment of MSCs' osteogenic differentiation while restoring skeletal homeostasis. However, several important limitations of this study should be explicitly acknowledged. First, while our In vitro data provide direct causal evidence that YGPs protect MSCs from oxidative damage and enhance osteogenic differentiation, In vivo histological and immunohistochemical results can't definitively prove that newly formed bone originates exclusively from MSCs. Lineage tracing—the gold standard for In vivo cell fate mapping—poses unique technical challenges for traditional Chinese medicine formulas, which contain multiple bioactive components exerting systemic effects on diverse cell types [46]. This complexity complicates isolating single‐lineage contributions using conventional transgenic models. Our observation of increased CD90^+^ COL1A1^+^ osteoprogenitors in bone marrow only indicates enhanced progenitor activity, not exclusive MSC‐derived bone formation. Second, we cannot fully exclude indirect effects of YGPs on other bone marrow cell types critical for bone homeostasis. Bone remodelling requires coordinated crosstalk between osteoblasts, osteoclasts, endothelial cells, and immune cells. While we focused on MSCs as the primary target, YGPs may also modulate osteoclast‐mediated resorption or endothelial angiogenesis—both essential for bone regeneration [47]. Consistent with the multi‐target nature of traditional Chinese medicine, future studies should explore additional targets and cell types involved in YGPs' bone‐protective effects.

## 5 Conclusion

In summary, YGPs reduce oxidative stress, activate the Nrf2/HO‐1 pathway, and restore MSCs osteogenic competence, culminating in improved bone microarchitecture in OVX mice. While our findings strongly support a central role for MSC protection and functional restoration in YGPs' osteoprotective effects, further studies are needed to confirm the In vivo cell origin of newly formed bone and to explore potential indirect effects on other bone cell types. Together with existing evidence for Nrf2's protective role in skeletal homeostasis, these findings support the further development of YGPs as a mechanism‐informed, redox‐modulating therapy for osteoporosis.

## Author Contributions

**Kairui Chen:** validation, investigation, writing – original draft, methodology, visualization, formal analysis. **Jingyuan Wen:** validation, investigation, writing – original draft, methodology. **Hongting Jin:** project administration, resources, supervision, validation, writing – review and editing. **Yungang Wu:** project administration, resources, funding acquisition, supervision, validation, writing – review and editing. **Liangyan Cheng:** validation, investigation, methodology. **Luwei Xiao:** project administration, supervision. **Jiali Chen:** supervision, validation, writing – review and editing. **Wenhua Yuan:** supervision, validation, writing – review and editing. **Qinwen Ge:** validation, investigation, methodology, writing – review and editing. **Qinghe Zeng:** validation, investigation, methodology. **Jiangyuan Liu:** conceptualization, investigation, writing – original draft, visualization, data curation, software. **Pinger Wang:** supervision, validation, writing – review and editing.

## Funding

This research has been partially supported by the National Natural Science Foundation of China (Grant no. 82374486) and the Zhejiang Chinese Medical University Postgraduate Scientific Research Fund Project (Y202351312).

## Conflicts of Interest

The authors declare no conflicts of interest.

## Supporting information


**Figure S1:** Differential expression analysis of GSE35956.
A. Volcano plot of differential expression in GSE35956.
B. Heatmap of differential expression in GSE35956.
C. Pathway analysis of GSE35956.
D‐F. GO analysis of GSE35956.

**Figure S2:** Differential expression analysis of GSE35958.
A. Volcano plot of differential expression in GSE35958.
B. Heatmap of differential expression in GSE35958.
C. Pathway analysis of GSE35958.
D‐F. GO analysis of GSE35958.

**Figure S3:** Differential expression analysis of GSE230665.
A. Volcano plot of differential expression in GSE230665.
B. Heatmap of differential expression in GSE230665.
C. Pathway analysis of GSE230665.
D‐F. GO analysis of GSE230665.

**Figure S4:** The most crucial enriched pathway.

**Figure S5:** Molecular docking verification.
The results showed that Benzoylmesaconine (−8.539 kcal/mol), Eudesmin (−7.597 kcal/mol), Geniposidic Acid−7.542 kcal/mol, Hokbusine A (−8.548 kcal/mol) and Senbusine C (−8.197 kcal/mol) exhibited significantly higher binding affinities to the Keap1 protein compared to other compounds, enabling them to competitively bind to the Kelch domain of Keap1, thereby releasing Nrf2 and activating downstream signalling pathways.

**Figure S6:** CCK‐8 assay to detect the viability of MSCs after Nrf2 siRNA transfection.
CCK8 results showed no significant differences between the NC siRNA group and the Nrf2 siRNA group at 24 h, 48 h, and 72 h.

**Table S1:** The detailed information of active ingredients contained in YGPs.
**Table S2:** The detailed information of active ingredients contained in drug serum.

## Data Availability

The data that support the findings of this study are available from the corresponding author upon reasonable request.
