# Supplementary figures and images for: Yougui Pills Alleviate Osteoporosis by Inhibiting Mesenchymal Stem Cell ROS Accumulation via the Nrf2/HO‐1 Pathway
Source: J Cell Mol Med. 2026 Jul 22;30(14):e71295. doi: 10.1111/jcmm.71295 (PMC13392220; doi:10.1111/jcmm.71295)

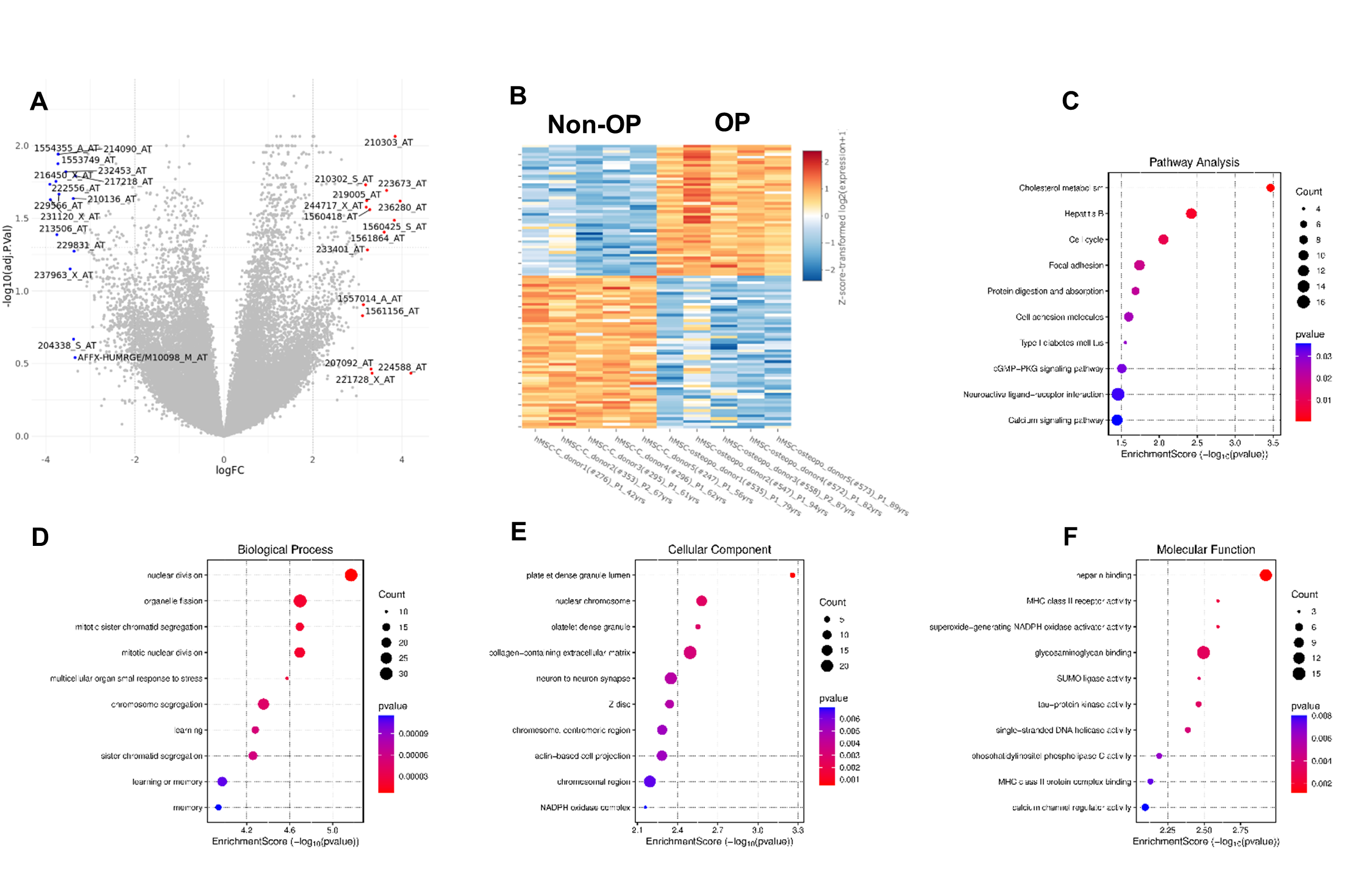

Supplement: Supplementary file 1 — Figure S1: Differential expression analysis of GSE35956. A. Volcano plot of differential expression in GSE35956. B. Heatmap of differential expression in GSE35956. C. Pathway analysis of GSE35956. D‐F. GO analysis of GSE35956. [file JCMM-30-e71295-s005.tif]

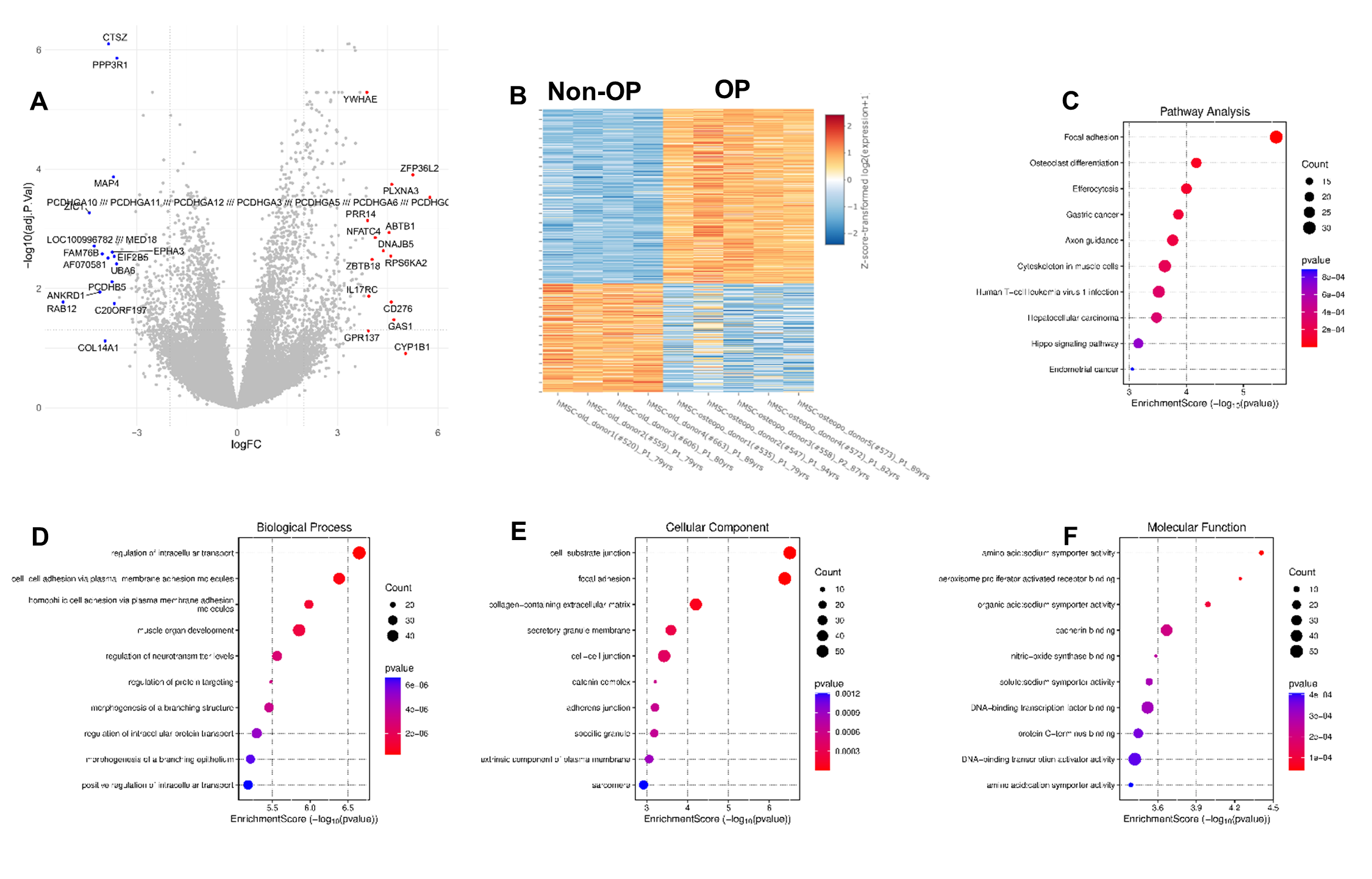

Supplement: Supplementary file 2 — Figure S2: Differential expression analysis of GSE35958. A. Volcano plot of differential expression in GSE35958. B. Heatmap of differential expression in GSE35958. C. Pathway analysis of GSE35958. D‐F. GO analysis of GSE35958. [file JCMM-30-e71295-s004.tif]

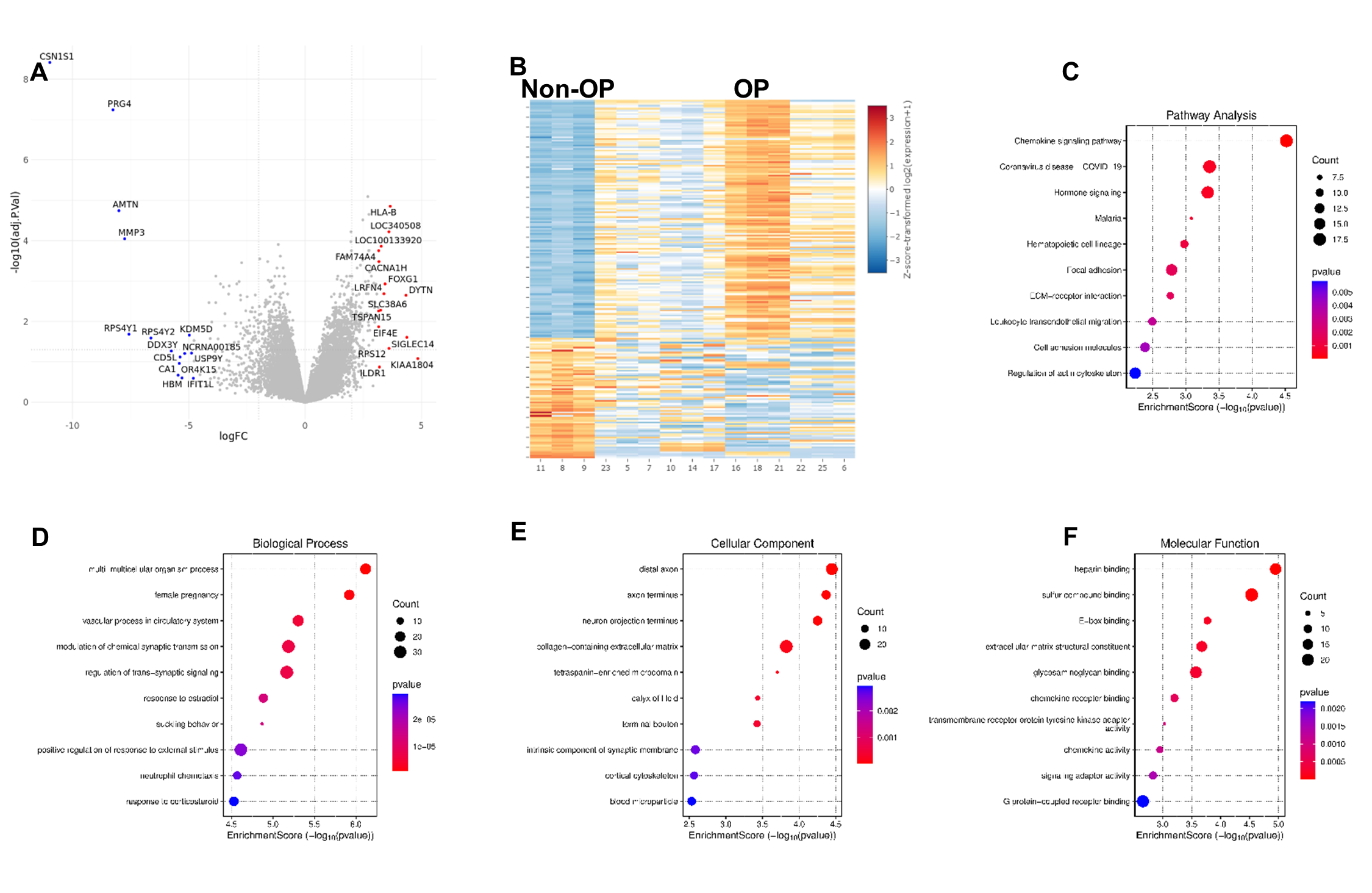

Supplement: Supplementary file 3 — Figure S3: Differential expression analysis of GSE230665. A. Volcano plot of differential expression in GSE230665. B. Heatmap of differential expression in GSE230665. C. Pathway analysis of GSE230665. D‐F. GO analysis of GSE230665. [file JCMM-30-e71295-s002.tif]

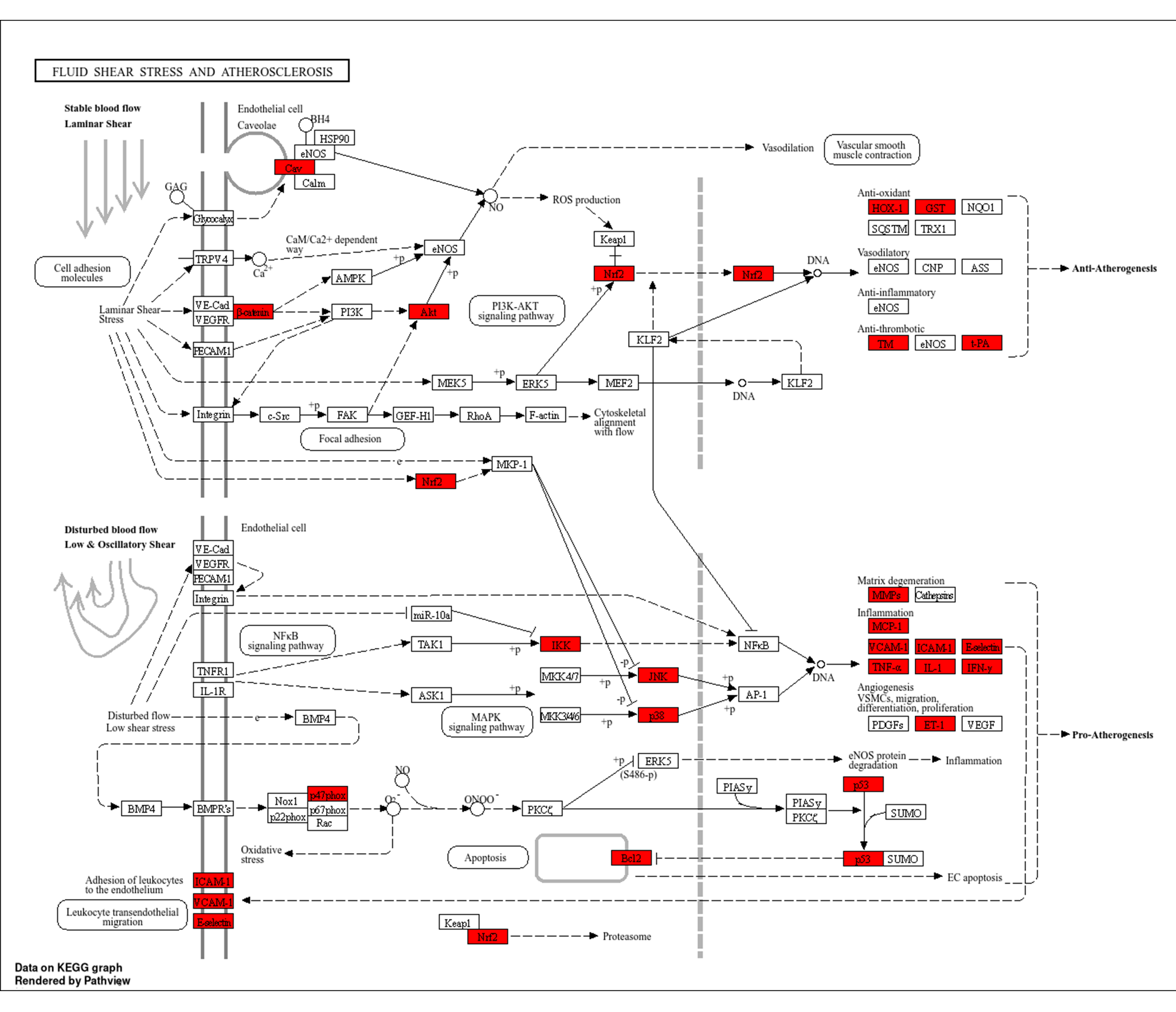

Supplement: Supplementary file 4 — Figure S4: The most crucial enriched pathway. [file JCMM-30-e71295-s001.tif]

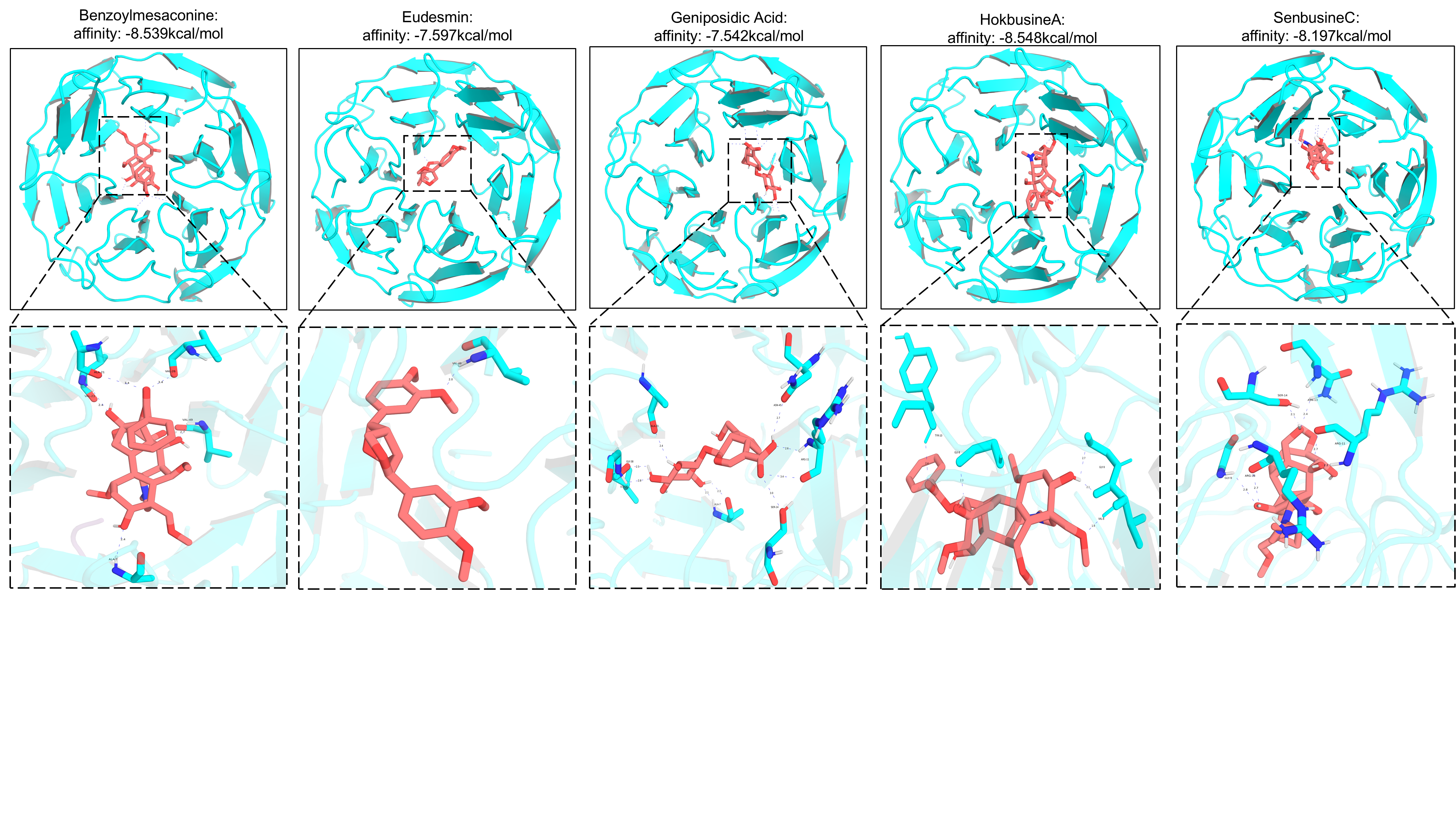

Supplement: Supplementary file 5 — Figure S5: Molecular docking verification. The results showed that Benzoylmesaconine (−8.539 kcal/mol), Eudesmin (−7.597 kcal/mol), Geniposidic Acid−7.542 kcal/mol, Hokbusine A (−8.548 kcal/mol) and Senbusine C (−8.197 kcal/mol) exhibited significantly higher binding affinities to the Keap1 protein compared to other compounds, enabling them to competitively bind to the Kelch domain of Keap1, thereby releasing Nrf2 and activating downstream signalling pathways. [file JCMM-30-e71295-s006.tif]

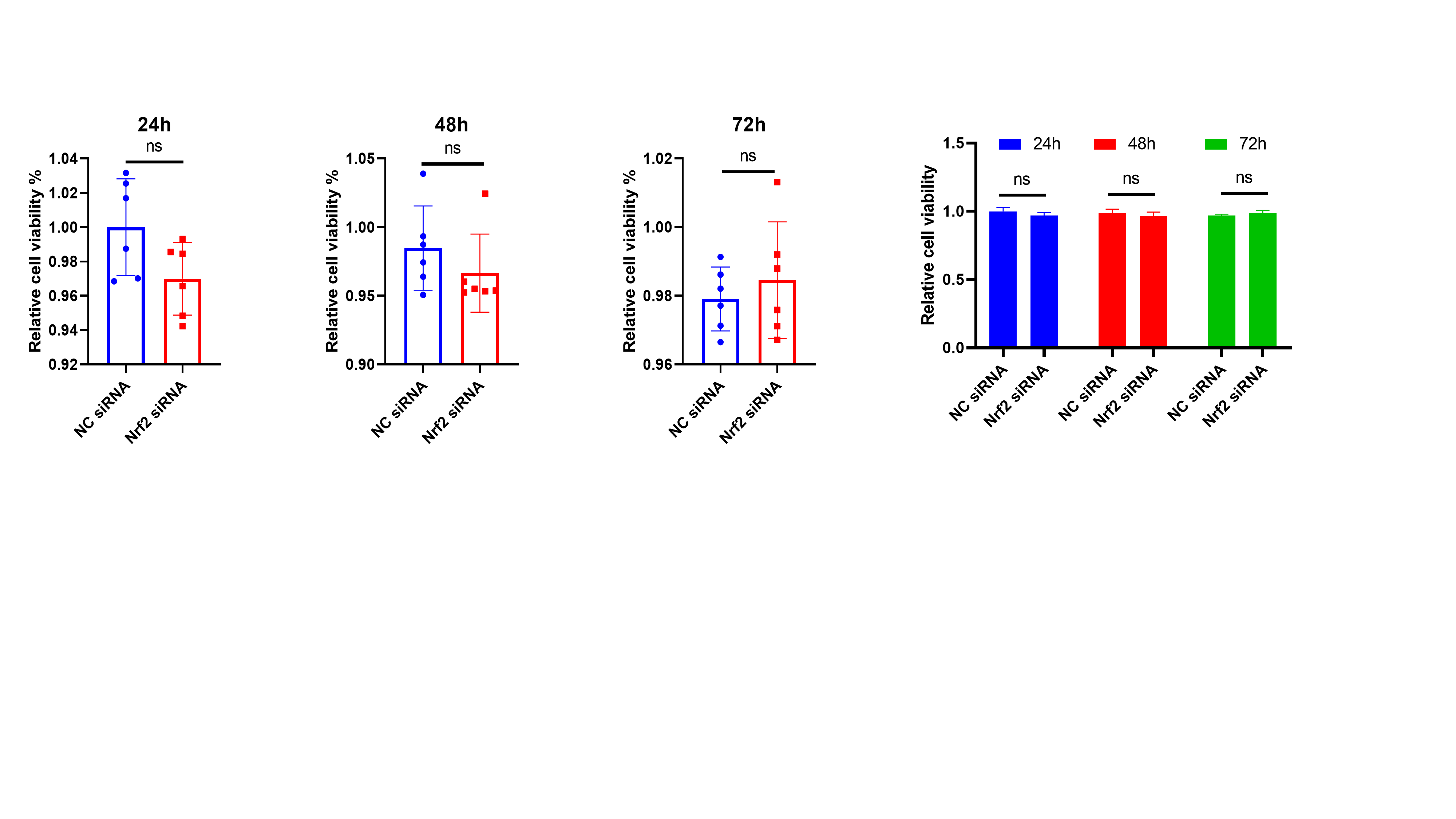

Supplement: Supplementary file 6 — Figure S6: CCK‐8 assay to detect the viability of MSCs after Nrf2 siRNA transfection. CCK8 results showed no significant differences between the NC siRNA group and the Nrf2 siRNA group at 24 h, 48 h, and 72 h. [file JCMM-30-e71295-s003.tif]
