# Supplementary material for: Yougui Pills Alleviate Osteoporosis by Inhibiting Mesenchymal Stem Cell ROS Accumulation via the Nrf2/HO‐1 Pathway
Source: J Cell Mol Med. 2026 Jul 22;30(14):e71295. doi: 10.1111/jcmm.71295 (PMC13392220; doi:10.1111/jcmm.71295)
Supplement: Supplementary file 7 — Table S1: The detailed information of active ingredients contained in YGPs. Table S2: The detailed information of active ingredients contained in drug serum. [file JCMM-30-e71295-s007.docx]

Supplementary Table 1

The detailed information of active ingredients contained in YGPs

| Peak no | Observed RT (min) | Component name | Formula | Observed m/z | Response | Adducts |
| --- | --- | --- | --- | --- | --- | --- |
| 1 | 9.5 | Benzoylmesaconine | C31H43NO10 | 590.2958 | 116883 | +H |
| 2 | 5.84 | Senbusine C | C24H39NO7 | 454.2799 | 23788 | +H |
| 3 | 0.78 | Isomaltose | C12H22O11 | 343.1237 | 14304 | +H |
| 4 | 0.73 | Geniposidic acid | C16H22O10 | 375.1277 | 13431 | +H, +Na, +K |
| 5 | 8.04 | Stearamide | C18H37NO | 322.2485 | 9294 | +K |
| 6 | 8.68 | 3,4-Dehydrolycopen-16-al | C40H52O | 549.4109 | 8716 | +H |
| 7 | 9.17 | Senkyunolide F | C12H14O3 | 207.1011 | 16640 | +H |
| 8 | 15.18 | Hexadecanamide | C16H33NO | 256.2629 | 25126 | +H |
| 9 | 15.37 | Eudesmin | C22H26O6 | 387.1802 | 32363 | +H, +Na, +K |
| 10 | 21.6 | 1,7-Bis(4-hydroxyphenyl)hepta-4,6-dien-3-one | C19H18O3 | 317.1147 | 14482 | +Na |

Supplementary Table 2

The detailed information of active ingredients contained in drug serum

| Peak no | Observed RT (min) | Component name | Formula | Observed m/z | Response | Adducts |
| --- | --- | --- | --- | --- | --- | --- |
| 1 | 8.96 | Benzoylmesaconine-C7H4O(cleavage)-H2O-H2+C6H8O6 | C30H43NO14 | 680.2367 | 10237 | +K |
| 2 | 9.41 | Isomaltose+2x(+O) | C12H22O13 | 397.094 | 29366 | +Na |
| 3 | 15.31 | Hexadecanamide | C16H33NO | 256.2627 | 45044 | +H |
| 4 | 17.71 | Senbusine C+2x(-H2O)+C2H2O | C26H37NO6 | 460.2702 | 5431 | +H |
| 5 | 19.32 | Hokbusine A-C7H4O(cleavage)+H2 | C25H43NO9 | 502.3025 | 7155 | +H |
| 6 | 19.71 | Geniposidic acid-C6H10O6(cleavage)-H2O+C10H15N3O6S | C20H25N3O9S | 522.098 | 5509 | +K |
| 7 | 20.48 | Stachyose-H2+C10H15N3O6S | C34H55N3O27S | 992.2704 | 4932 | +Na |
| 8 | 23.05 | Dimethyl D-malate-CH2(cleavage)+H2O+O+C10H15N3O6S | C15H25N3O13S | 526.0731 | 21667 | +K |
| 9 | 32.96 | Senkyunolide F+H2+C10H15N3O6S | C22H31N3O9S | 536.1653 | 2551604 | +Na |
